# Structural Relationships to Efficacy for Prazole‐Derived Antivirals

**DOI:** 10.1002/advs.202308312

**Published:** 2024-03-06

**Authors:** David A. Nyenhuis, Susan Watanabe, Rebecca Bernstein, Rolf E. Swenson, Natarajan Raju, Venkata R. Sabbasani, Chandrasekhar Mushti, Duck‐Yeon Lee, Carol Carter, Nico Tjandra

**Affiliations:** ^1^ Biochemistry and Biophysics Center NHLBI NIH 50 South Drive, Bld 50, Rm 3503 Bethesda MD 20892 USA; ^2^ Department of Microbiology and Immunology Renaissance School of Medicine Stonybrook University Life Sciences Bldg, Rm 248 Stonybrook NY 11790 USA; ^3^ Chemistry and Synthesis Center NHLBI NIH 9800 Medical Center Drive, Bldg B, #2034 Rockville MD 20850 USA; ^4^ Biochemistry Core Facility NHLBI NIH Bethesda MD 20892 USA

**Keywords:** antiviral agents, biophysics, drug design, NMR spectroscopy, protein modifications

## Abstract

Here, an in vitro characterization of a family of prazole derivatives that covalently bind to the C73 site on Tsg101 and assay their ability to inhibit viral particle production is presented. Structurally, increased steric bulk on the 4‐pyridyl of the prazole expands the prazole site on the UEV domain toward the β‐hairpin in the Ub‐binding site and is coupled to increased inhibition of virus‐like particle production in HIV‐1. Increased bulk also increased toxicity, which is alleviated by increasing flexibility. Further, the formation of a novel secondary Tsg101 adduct for several of the tested compounds and the commercial drug lansoprazole. The secondary adduct involved the loss of the 4‐pyridyl substituent to form an irreversible species, with implications for increasing the half‐life of the active species or its specificity toward Tsg101 UEV. It is also determined that sulfide derivatives display effective viral inhibition, presumably through cellular sulfoxidation, allowing for delayed conversion within the cellular environment, and identify SARS‐COV‐2 as a target of prazole inhibition. These results open multiple avenues for the design of prazole derivatives for antiviral applications.

## Graphical abstract

Characterization of a family of covalent prazole derivative adducts with the Tsg101 UEV domain finds roles for bulk and flexibility in antiviral efficacy and cytotoxicity, while opening avenues for further development of prazole‐based antiviral therapeutics that target Tsg101.

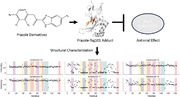

## 1 Introduction

Prazoles are benzimidazole derivatives marketed as stomach‐acid reducers, owing to their ability to inhibit the H+/K+ ATPases (proton pumps) of the parietal cells in the stomach epithelium. In this context, the prazole compound undergoes an acid‐catalyzed conversion from the pro‐drug to an active cyclic sulfenamide or sulfenic acid form, which can undergo covalent attachment via disulfide linkage to cysteine residues on the proton pump.^[^ ^1^, ^2^ ^]^ The formation of a covalent adduct to the ATPase, together with the ability to attach to multiple cysteines on the protein's surface make prazoles potent inhibitors of acid efflux.^[^ ^2^ ^]^ Additionally, the conversion to the active species is acid‐catalyzed, leading to the accumulation of active drugs at the desired site of action.^[^ ^2^ ^]^

The ability to administer a prodrug form of the compound, which is contextually activated, together with the formation of covalent adducts with protein cysteine residues, have recently made prazoles an attractive class for anticancer,^[^ ^3^, ^4^ ^]^ antiprotozoal,^[^ ^5^ ^]^ antibiotic,^[^ ^6^ ^]^ and antiviral applications.^[^ ^7^, ^8^, ^9^, ^10^ ^]^ Beyond these, a large‐scale target trial emulation study of Alzheimer's disease data identified two prazoles in the top five candidates repurposable for Alzheimer's disease.^[^ ^11^ ^]^ In the viral context, we and others have recently shown that commercial prazoles can inhibit the egress of several viral targets: human immunodeficiency virus type‐1 (HIV‐1), herpes simplex virus‐1 and ‐2, Epstein‐Barr virus, Mayaro virus, and Ebola virus^[^ ^7^, ^8^, ^10^ ^]^ Here, the target is Tsg101, a member of the Endosome Sorting Complex Required for Trafficking (ESCRT)‐I complex.^[^ ^7^ ^]^ The ESCRT complexes (0, I, II, III) constitute a modular membrane budding machinery, responsible for a range of membrane budding and remodeling events in the cell, including multivesicular body (MVB) formation, membrane repair, cytokinetic abscission, autophagy, and exosome release.^[^ ^12^ ^]^ Tumor susceptibility gene 101 (Tsg101) is a member of the ESCRT‐I complex, where it functions as both an adaptor to other ESCRT members and a Ubiquitin (Ub) sensor. This is accomplished by its modular architecture; with an N‐terminal Ubiquitin E2 variant (UEV) domain followed by a proline‐rich region, a coiled‐coil domain, and a C‐terminal steadiness box. Compared to canonical E2s, the Tsg101 UEV domain lacks the catalytic cysteine yet retains ubiquitin affinity, making it a Ub sensor or chaperone rather than an enzyme.^[^ ^13^, ^14^, ^15^ ^]^ It also contains a distinct P[T/S]AP site, which interacts with the ESCRT‐0 member HRS and other endogenous factors.^[^ ^13^, ^16^ ^]^ The proline‐rich region downstream of the UEV domain contains an EABR domain interacting region, which facilitates Tsg101 recruitment by CEP55 to sites of cytokinetic abscission,^[^ ^17^ ^]^ while the coiled‐coil facilitates further interactions with ESCRT‐1 factors^[^ ^18^, ^19^ ^]^ and the steadiness box contains lysine residues that are polyubiquitylated by regulatory E3 ligases, including Tal, to control the steady‐state levels of Tsg101.^[^ ^20^ ^]^

The ability of the ESCRT complex to facilitate membrane egress is frequently hijacked by retroviruses, including human immunodeficiency virus type‐1 (HIV‐1), for egress from the nuclear envelope, the plasma membrane, or for encapsulation into multivesicular bodies within the endosome, which can be rerouted for exocytosis.^[^ ^12^, ^21^ ^]^ To recruit the ESCRT machinery, retroviruses typically encode PPXY, PTAP, or YXXL motifs to recruit ESCRT‐associated factors,^[^ ^22^ ^]^ where the C‐terminal P6 region of the HIV‐1 Gag polyprotein, specifically, contains a PTAP motif for interaction with a distinct pocket on the Tsg101 UEV domain.^[^ ^13^ ^]^ While the ESCRT system is responsible for the formation and release of endogenous exosomes from the PM, HIV‐1 particles are assembled directly on the PM, rather than in MVBs. This requires the Gag‐Tsg101 complex to be present at the PM, which may be driven by the membrane binding ability of the matrix domain in HIV‐1 Gag.^[^ ^23^ ^]^

In this context, prazoles bind to C73 in the middle of the Ub‐binding site of the Tsg101 UEV domain, sterically inhibiting the Ub‐Tsg101 interaction.^[^ ^7^ ^]^ While the Ub and P[T/S]AP sites on the UEV domain are spatially distinct, their close proximity and the presence of P[T/S]AP motifs in the late domains of many viruses complicates the functional interpretation of prazole‐inhibition of viral egress. Still, blockage of the Ub‐Tsg101 interaction appears to be the dominant mode of inhibition.^[^ ^7^ ^]^ In support of this, our prior NMR characterization of prazoles bound to C73 on Tsg101 found minimal perturbation within the PTAP pocket.^[^ ^7^ ^]^ Prazoles also arrest viral assembly at an earlier, visually distinct point of assembly than seen for depletion of Tsg101 or mutation of the viral P[T/S]AP motif.^[^ ^7^ ^]^ Additionally, the dengue virus, a flavivirus that has a PTAP sequence for Tsg101 recruitment but is not impacted by mutation of the Ub‐binding site in Tsg101 was also not sensitive to prazole treatment.^[^ ^8^, ^24^ ^]^

Screening of commercially available prazoles found that larger compounds (rabeprazole and ilaprazole) are generally more effective at inhibiting viral egress, where this may stem from more rapid conversion to the active form, or from the larger prazole adduct on the Tsg101 UEV domain providing a more effective blockade of Ub‐binding.^[^ ^9^, ^25^ ^]^ To test this further, here we screened 20 prazole‐derivatives, both in vitro for differences in their binding to Tsg101, and in the cell, where we identified 10 compounds as having < 5 µm IC50 against HIV‐1 and identified SARS‐COV‐2 as a further target of prazole‐based inhibition. Structurally, we find that increased bulk on the 4‐pyridyl substituent of the prazole expands its impact on the UEV domain toward the β‐hairpin in the Ub‐binding site, which is coupled to increased inhibition of virus‐like particle (VLP) production in HIV‐1. This modification also increased toxicity, however, which was alleviated by increasing flexibility. Further, seven of the tested compounds and the commercial compounds lansoprazole and esomeprazole led to secondary adducts with Tsg101 UEV. As shown for lansoprazole, secondary adducts involved loss of the 4‐pyridyl substituent and were irreversible, with implications for increasing the half‐life of the active species or its specificity toward the Tsg101 UEV domain.

Finally, prazoles are known to be metabolized in the liver by members of the cytochrome P450 (CYP) family, namely CYP3A4 and CYP2C19.^[^ ^2^, ^26^ ^]^ Metabolism primarily involves hydroxylation to hydroxyprazoles, or sulfoxidation to prazole‐sulfone derivatives.^[^ ^26^ ^]^ We speculated that reduced sulfide derivatives may be similarly oxidized in‐cell to the sulfoxide pro‐drug, altering the active profile of the drug and the site of activation. As expected, a sulfide derivative of one of our tested compounds displayed no significant chemical shifts or evidence of labeling in vitro by NMR, while displaying similar antiviral activity to the parent prazole‐sulfoxide in VLP assays. Cellular activity of the sulfide derivative was sensitive to N‐acetyl‐cysteine‐treatment, indicating that it was still dependent on cysteine attachment to Tsg101. Thus, the use of sulfide derivatives as prodrugs may be an effective avenue to alter drug targeting and kinetics for antiviral applications. These insights expand the breadth of options available for the design and optimization of the prazole compound family for antiviral applications.

## 2 Results and Discussion

### 2.1 Structural Comparison of Commercial and Tested Prazole‐Derivatives

**Figure 1:**
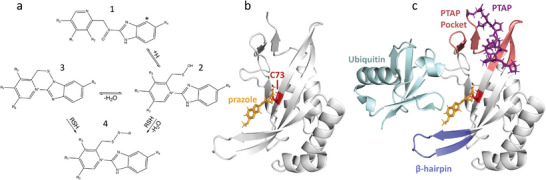
Overview of prazoles in the context of Tsg101. A) Prazole scaffold and scheme. Positions on the prazoles pro‐drugs (1) are highlighted (R1‐R4), which are substituted in the commercial prazoles or derivatives tested in this work. Our tested derivatives differ primarily in the group at the R3 position on the pyridine ring. The * position in (1) is replaced by Nitrogen in tenatoprazole. The prazole pro‐drug undergoes a ring conversion to active sulfenic acid (2) or tetracyclic sulfenamide (3) forms, either of which can form a disulfide with cysteine residues of a target protein (4) in the cell. B) Prazoles (orange) form an adduct with C73 (red) on the Tsg101 UEV domain (gray), which is shown for our previously determined pose with tenatoprazole (PDB ID: 5VKG).^[^ ^7^ ^]^ (C) The prazole adduct forms in a pocket on the Tsg101 UEV surface near the ubiquitin (cyan) binding site, which includes the unique β‐hairpin extension (blue). The UEV domain also contains a pocket (pink) capable of binding P[T/S]AP signals (purple), which recruit it to endogenous ESCRT machinery and are used by retroviral Gag proteins to hijack Tsg101. The ubiquitin binding mode is taken from an overlay of PDB ID: 1S1Q, while the PTAP binding mode is taken from the overlay of PDB ID: 1M4Q.

The prazole scaffold is shown in **Figure** **1**a, where variations in the R_1_ group on the benzimidazole, and in the R_2_, R_3_, and R_4_ positions on the pyridine ring are present in commercial prazoles and the derivatives explored here. Prazoles are pro‐drugs (Figure 1a,1), which undergo a ring conversion to active sulfenic acid (2) or tetracyclic sulfenamide (3) forms, which form disulfide linkages to cysteine residues (4) in the cell.^[^ ^2^ ^]^ On the Tsg101 UEV domain (Figure 1b, gray), the prazole (orange) attaches to C73 (red), where we previously determined the orientation of the resulting adduct for tenatoprazole.^[^ ^7^ ^]^ Contextually, the prazole adduct sits in a pocket on the UEV surface near the ubiquitin‐binding site (Figure 1C, cyan), where in particular it may interfere with the β‐hairpin extension (blue), a distinct feature of the Tsg101 Ub‐binding surface.^[^ ^13^ ^]^ The UEV domain also contains a pocket (pink) capable of binding P[T/S]AP signals (purple), which recruit it to endogenous ESCRT machinery and are used by retroviral Gag proteins to hijack Tsg101.^[^ ^13^ ^]^

**Figure 2:**
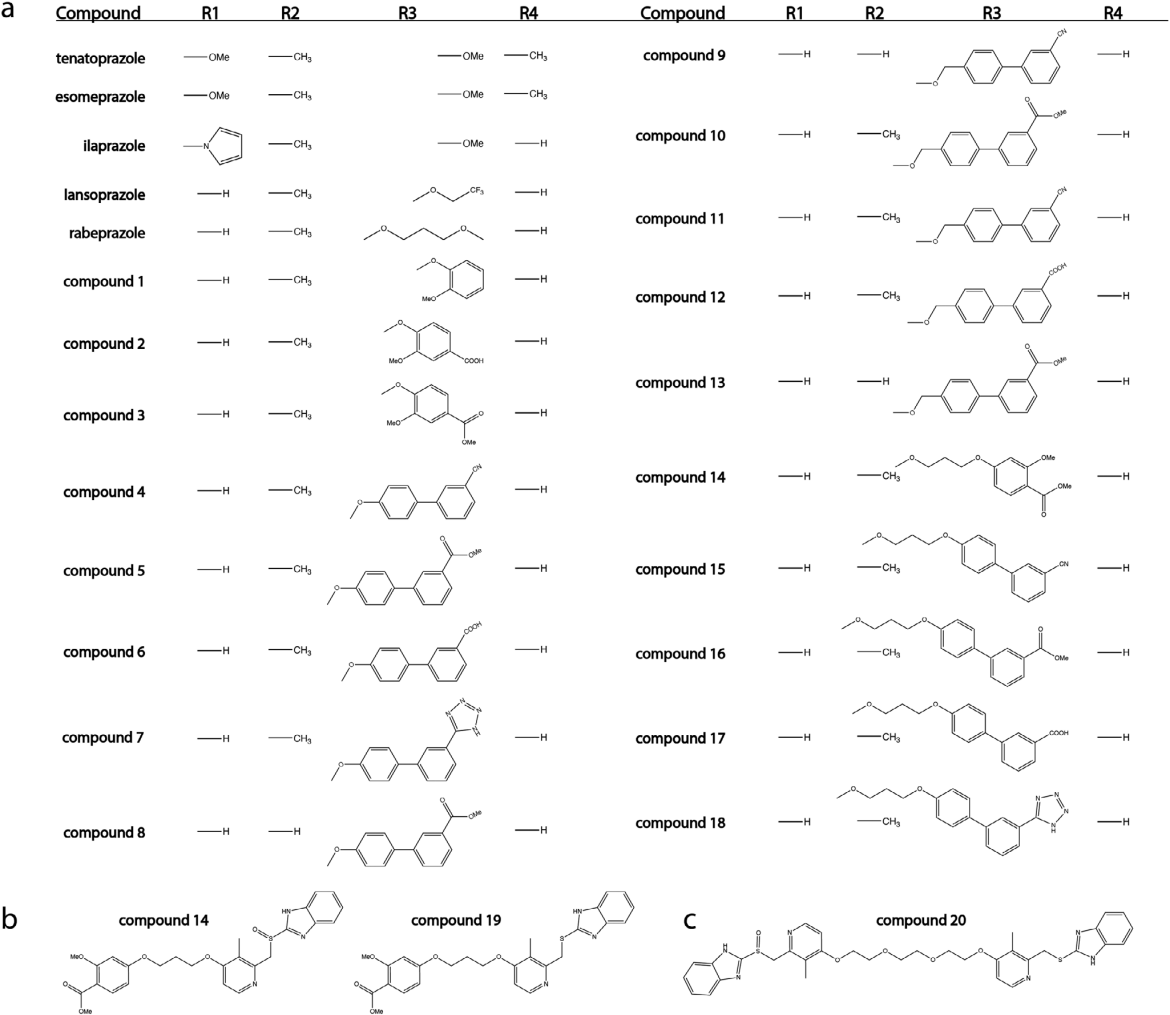
Summary of the variations in all tested prazoles. A) Compound names and the corresponding R1‐R4 groups. Only two commercial prazoles (tenatoprazole and esomeprazole) contain R4 substitutions on the pyridyl ring. Three (tenatoprazole, esomeprazole, ilaprazole) also contain R1 substitutions on the benzimidazole. The tested derivatives follow the general scheme of lansoprazole or rabeprazole, with variations in the R3 group. Derivatives are ordered by the length of the R3 ether linkage and bulk. Nine derivatives have the shortest linker length, where three (1, 2, 3) are phenyl derivatives and the other six are biphenyl. A further four compounds (10, 11, 12, 13) have an additional carbon in the R3 linker and are all biphenyl derivatives. The next five have the extended linker taken from rabeprazole where one (14) is a phenyl derivative and the others (15, 16, 17, 18) are biphenyls. B) In addition to compound 14, its sulfide derivative (19) was used to screen whether compound 19 could be converted to the parent sulfoxide within the cell. (C) A final compound (20) is a rabeprazole derivative dimerized through the linker, with one sulfoxide and one sulfide.

In addition to five commercial prazoles, we synthesized and characterized 20 further prazole derivatives (**Figure** **2**) by in vitro and cellular assays. Most compounds (Figure 2a) are based on the minimal scaffold in lansoprazole or rabeprazole, where the benzimidazole R_1_ is hydrogen, the pyridyl R_2_ and R_4_ positions are methyl and hydrogen, respectively, and the 4‐pyridyl position (R_3_) is variable. The smallest compounds (1‐3) have ether R_3_ groups based on methoxy derivatives of benzene (1), benzoic acid (2) and benzoate (3). The second group of compounds (4‐9) is biphenyl based, with meta‐substitutions on the distal ring. These are nitrile (4, 9), methyl acetate (5, 8), carboxy (6), and tetrazole (7). Compounds 8 and 9 additionally substitute hydrogen for methyl at R_2_. This second group was designed to increase bulk on the UEV surface within the Ub site.

The third group (10‐13) was derived from the second, with increased flexibility through a single carbon extension in the R_3_ linker. Here, the biphenyl was functionalized with methyl acetate (10, 13), nitrile (11), and carboxy (12), where compound 13 again has hydrogen at R_2_. The fourth group of derivatives (14‐18) further increased flexibility using the extended R_3_ linker of the rabeprazole parent. Compound 14 has a smaller methyl benzoate group attached to the rabeprazole linker (as in 3), while compounds 15–18 have meta substitutions on the distal biphenyl ring of nitrile (15), methyl acetate (16), carboxy (17), and tetrazole (18). Compound 19 is derived from 14 (Figure 2b), where the core prazole sulfoxide is reduced to the corresponding sulfide, which was expected to prevent prodrug activation and subsequent attachment to the Tsg101 UEV. Finally, compound 20 is a dimeric derivative of rabeprazole, with one active sulfoxide and one inactive sulfide (Figure 2c).

### 2.2 Tracking Prazole‐Tsg101 Adduct Formation with Solution NMR

Much of the work on prazoles as therapeutic agents in areas outside their current role as proton pump inhibitors have focused on the activation of the prazole pro‐drug in isolation. This regime is highly relevant to the parietal cell context, where the pH may be < 2,^[^ ^27^ ^]^ and where the primary target (the gastric H+/K+ ATPase) presents extracellular cysteines. In the antiviral context, however, the primary target (Tsg101) is present in both cytoplasmic and membrane‐associated contexts,^[^ ^20^, ^28^ ^]^ where the latter is partitioned between endosomal and plasma membrane‐associated structures.^[^ ^28^ ^]^ In this regard, pro‐drug rearrangement and attachment may reasonably be expected to occur at cytosolic pH, or in the weakly acidic conditions of the early endosome (pH 5.5‐6.5),^[^ ^29^ ^]^ far from the optimum for prazole derivatives.

**Figure 3:**
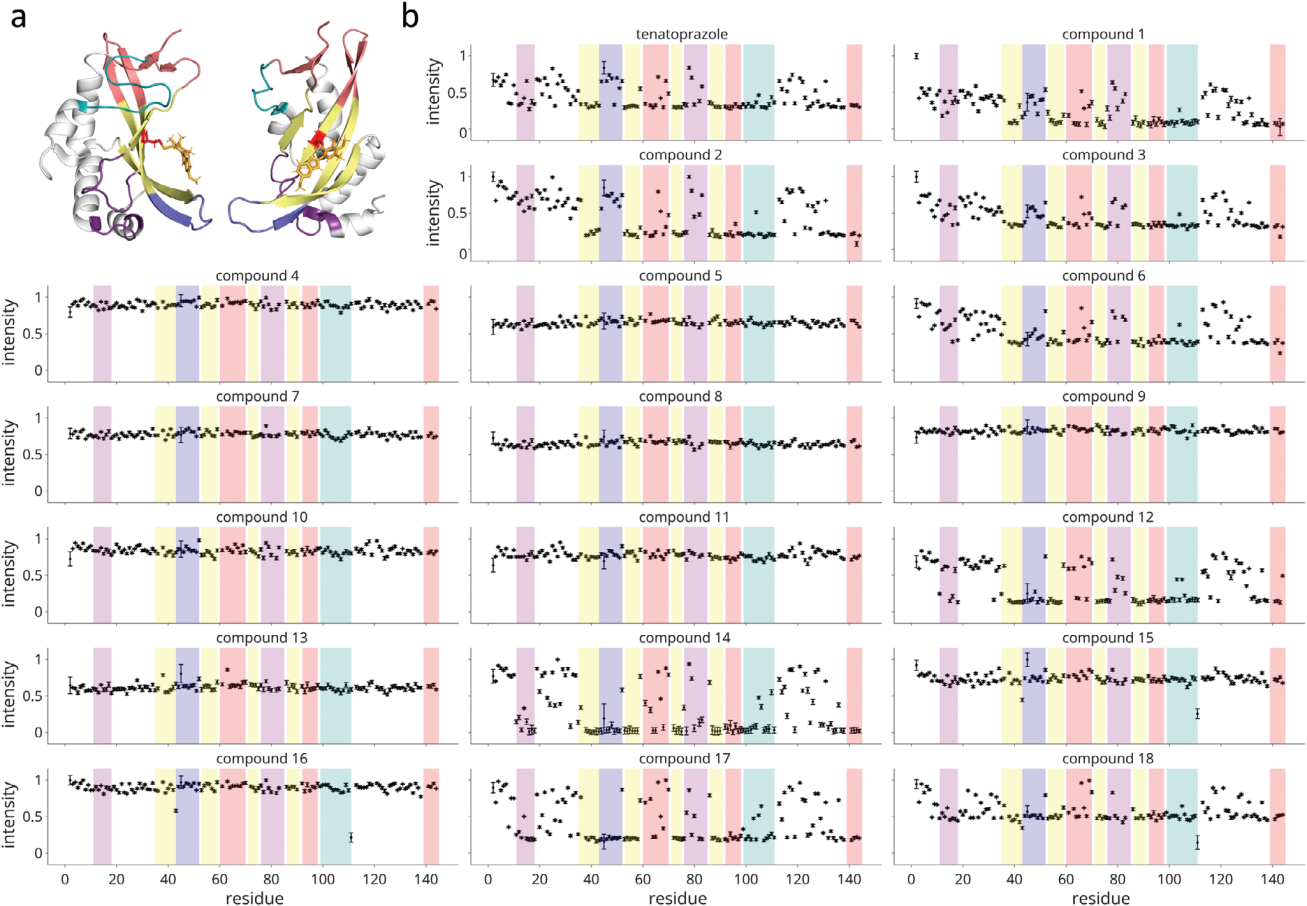
Intensity loss profiles 7 h post‐addition of prazole for tested derivatives show the impact of steric bulk on the prazole‐UEV adduct. A) Two views of the Tsg101 UEV domain, highlighting the prazole (orange) on C73 (red) (PDB ID: 5VKG), the residues in the immediate vicinity in yellow, and accessory regions. The beta‐hairpin extension, which forms part of the Ub‐binding site is shown in blue, and two unstructured regions that contact it are shown in purple. On the opposite face, the P[T/S]AP motif binding pocket is shown in pink, and the proximal vestigial active site region is shown in teal. B) Normalized intensity profiles 7 h after the addition of prazole for tenatoprazole and eighteen tested derivative compounds. The regions shown in (A) are again colored to show strongly and weakly impacted regions. Intensities are the percentage of the free Tsg101 chemical shift remaining at each site. Tenatoprazole and the smallest derivatives (1‐3), which have phenyl substituents at the R3 position, show similar profiles with strong impacts in the immediate vicinity (yellow) of the prazole‐binding pocket. They also show moderate impact in the PTAP pocket (pink) and the vestigial active site (teal), and only mild impact in the beta‐hairpin. Many of the larger derivatives (4,5,7,8,9,10,11,13,16) show minimal attachment, either reflective of limited conversion or poor solubility. Those larger derivatives that appear to show significant attachment (6, 12, 14, 17, 18) again have a strong impact in the prazole‐binding site but show a significant loss at the beta‐hairpin extension, rather than the PTAP pocket, indicating that they may be more strongly block Ub binding. Error bars are derived from the noise level of the source spectra obtained using the Estimate Noise Tool in NMRPipe and NMRDraw for each spectrum.

We reasoned that this would increase the importance of looking at prazole‐protein adduct formation and may lead to unexpected structural modifications. Further, the prazole‐Tsg101 complex has increased solubility relative to the free prazole, where many of the tested compounds had limited solubility due to their bulky R_3_ modifications. We first tried a screen of tenatoprazole and 18 prazole derivatives, with the labeling of Tsg101 followed over time using successive 2D heteronuclear single quantum correlation (HSQC) experiments. Formation of the Tsg101‐prazole adduct at C73 causes chemical shifts in the slow‐exchange regime, allowing us to follow labeling by reductions in the intensity of the free Tsg101 UEV peak positions. This is shown in **Figure** **3** for the tested compounds at pH 5.8, 7 h after the addition of prazole, comparable to the pretreatment time (6 h) we found to be optimal for inhibition of HIV‐1 viral particle egress by commercial prazoles.^[^ ^7^ ^]^

Residue C73 is in the central β‐sheet of the UEV domain, where attachment of the core prazole structure (Figure 3a, orange) is expected to primarily impact residues in this vicinity (Figure 3a, yellow). Prazole compounds appear to sterically occlude Ub binding to the UEV domain, an interaction that involves several determinants in the β‐hairpin extension (blue), where greater impact in this region should correlate to reduced Ub‐binding. Opposite the Ub site, the UEV domain can also bind to P[T/S]AP motifs at a defined pocket (pink), which is coupled to the vestigial active site (teal). Commercial prazoles were previously found to have little impact in this region, although this could be altered for the bulkier derivatives tested here.^[^ ^7^, ^8^ ^]^

At the 7‐h point (Figure 3b), tenatoprazole causes significant intensity loss at all residues in the vicinity of C73, as expected and consistent with the previously reported chemical shifts.^[^ ^7^ ^]^ There is a moderate reduction in the PTAP pocket and vestigial active site, but only minor changes in the β‐hairpin extension and proximal regions (purple) underneath the β‐hairpin. The phenyl derivatives (compounds 1–3) show comparable or greater signal loss in the same regions. The larger derivatives are bifurcated between compounds with minimal intensity reduction (compounds 4,5, 7–9, 10, 11, 13, 16) versus clear attachment (6, 12, 14, 17, 18). The latter includes three (6,12,17) carboxy functionalized compounds, the single‐ring derivative with a rabeprazole linker (14), and a tetrazole (18), implying favorable solubility. Compared to tenatoprazole and compounds 1–3, these larger derivatives more effectively perturb the β‐hairpin region (particularly 12 and 17), with a reduced impact in the PTAP binding pocket. This favoring of the Ub‐binding site over the PTAP site was expected to represent a favorable profile for the larger derivatives.

We next looked specifically at the labeling site (C73) over a 24‐h period (Figure S1, Supporting Information). The same bifurcation between effective and ineffective attachment is seen, with fits to a simple exponential decay given in **Table** **1**. For the smallest derivatives (1‐3), the apparent rate was in the order of size (1 > 2 > 3). Similarly, for those compounds having a rabeprazole‐derived linker, the single‐ring derivative (14) had the fastest apparent attachment, while for the biphenyls the fastest was the carboxy (17), followed by tetrazole (18), nitrile (15), and finally methyl acetate (16). Interestingly, compound 12, which differs from compound 6 only in its extended linker, displayed a faster apparent rate of attachment. While limited in coverage by solubility, these results indicate a rate preference for smaller derivatives, and for greater linker flexibility at the R_3_ position. The fastest apparent rate was seen for compound 14, a phenyl derivative with the highly flexible rabeprazole linker.

**Table 1:** Summary Parameters for the in vitro characterization of Tsg101‐prazole adduct formation for tested commercial Prazoles and Derivatives. All Adduct masses are the observed mass delta by LC/MS. For the initial characterization in NMR buffer at mildly acidic conditions, rates, and final intensities were obtained from an exponential decay fit to the intensity at C73 in the UEV domain, as shown in Figure S1 (Supporting Information). Efficiencies in the neutral PBS buffer were taken from LC/MS after 48 h. Errors in the NMR parameters for loss of intensity at C73 (k, Final Intensity) were taken from the covariance matrix during fitting, where sigma was assumed to come from the noise level of the source spectra, estimated using the Estimate Noise function in NMRPipe and NMRDraw. The % labeling values and % secondary adduct estimated by LC / MS have an error of ≈10%, measured across runs using lansoprazole (high labeling) and Compound 1 (low labeling) as representative compounds.

Compound	MW	Pred. Mass Adduct	k, pH 5.8	Final Intensity [%], pH 5.8	Labeling pH 7.4 [%]	Adducts	Adduct Mass 1	Adduct Mass 2	% Secondary Adduct
**Tenatoprazole**	346.4	328.4	0.28 ± 0.01	23.4 ± 0.01	0.8	1	329.2		
**Lansoprazole**	369.36	351.36			>0.9	2	351.2	251.3	0.7
**Rabeprazole**	359.44	341.44			>0.9	1	342.9		
**Ilaprazole**	366.44	348.44			>0.9	1	349.6		
**Esomeprazole**	345.417	327.417			>0.9	1		295.9	>0.9
**Compound 1**	393.46	375.46	0.48 ± 0.01	4.3 ± 0.01	0.2	2	375.4	251.7	0.5
**Compound 2**	437.446	419.446	0.24 ± 0.01	10.1 ± 0.01	0.1	2	419.6	251.7	0.4
**Compound 3**	451.5	433.5	0.13 ± 0.01	1.8 ± 0.01	0.8	2	433.5	251.8	>0.9
**Compound 4**	464.13	446.13	0.07 ± 0.02	79.5 ± 0.03	>0.9	1		251.8	>0.9
**Compound 5**	497.14	479.14	0.48 ± 0.04	66.9 ± 0.01	0	0			
**Compound 6**	483.54	465.54	0.18 ± 0.01	22.5 ± 0.01	>0.9	2	465.8	251.8	>0.9
**Compound 7**	507.57	489.57	0.42 ± 0.05	78.7 ± 0.01	>0.9	2	489.8	251.9	>0.9
**Compound 8**	483.54	465.54	0.38 ± 0.03	64.0 ± 0.01	0	0			
**Compound 9**	450.54	432.54	0.18 ± 0.02	79.6 ± 0.01	0	0			1
**Compound 10**	511.16	493.16	0.11 ± 0.02	79.8 ± 0.01	0.5	1	494.1		
**Compound 11**	478.15	460.15	0.07 ± 0.01	54.3 ± 0.03	>0.9	1	461.3		
**Compound 12**	497.57	479.57	0.40 ± 0.01	11.5 ± 0.01	>0.9	1	480.1		
**Compound 13**	497.14	479.14	0.39 ± 0.03	62.1 ± 0.01	0.4	1		237.9	>0.9
**Compound 14**	512.523	494.523	0.87 ± 0.03	1.0 ± 0.01	0.6	1	492.2		
**Compound 15**	523	505	0.12 ± 0.01	65.1 ± 0.01	0.5	1	505		
**Compound 16**	556	538	0.06 ± 0.01	68.2 ± 0.01	0.4	1	538		
**Compound 17**	542	524	0.30 ± 0.01	13.4 ± 0.01	0.4	1	524		
**Compound 18**	566	548	0.26 ± 0.01	40.2 ± 0.01	>0.9	1	548		
**Compound 19**	496.5	478.5			0	0			
**Compound 20**	672.82	654.82			0.5	1	655.4		

### 2.3 Bimodal Structural Grouping of Prazole‐Tsg101 Adducts

Incomplete labeling and limited solubility complicated our ability to collect triple resonance experiments and thus assign many of the prazole adducts. Instead, we opted for a global analysis of the collected HSQC spectra using principal component analysis (PCA), where dimensional reduction using PCA was expected to make it easier to spot compounds with similar effects or pose on the UEV surface. All spectra used in the intensity‐based analysis of the 18 compounds shown in Figures 3 and Figure S1 (Supporting Information) were used in the PCA, following normalization to their most intense peak. Due to the number of spectra, we opted to use the 2D NMR data directly in the PCA, as in several recent approaches.^[^ ^30^, ^31^, ^32^, ^33^, ^34^ ^]^ Plots of the first, second, and third principal components (PCs) are shown in Figure S2 (Supporting Information). Both PC1 and PC2 seem to report primarily on labeling, matching the trend for intensity loss at C73. By PC1 value, compound 14 is followed by four compounds (1, 3, 12, 17), then by three more (2, 6, 18) which are comparable to tenatoprazole.

Contrastingly, PC3 splits into three potential groups. Low PC3 values include the rabeprazole derivatives, where 17 and 18 have large PC2 values and 15 and 16 smaller ones. Values near zero include compounds 12, 14, and 15 which have the intermediate linker length and biphenyls. Large PC3 values include tenatoprazole, the three smallest derivatives (1, 2, 3), and compound 6, which had the short linker and bulky biphenyl. Separation based on PC3 appears tied to chemical shifts rather than intensity differences, as compounds 17 and 18 display different rates and behavior at C73 with comparable PC3 values. PC3 coordinate is also in reasonable agreement with the regional intensity losses 7 h after prazole addition. There, the separation was between tenatoprazole plus the smallest compounds (1, 2, 3) versus five bulkier derivatives (6, 12, 14, 17, 18); diverging from PC3 only in the grouping of compound 6. Together, the global analysis confirmed the apparent attachment rates seen by following the intensity loss at residue C73 in the Tsg101 UEV domain and separated the derivatives into two classes, based apparently on R_3_ size. This correlated to the intensity trends at 7 h post addition, indicating that lower PC3 coordinates might be ascribed to greater bulk and the desired greater impact at the Ub‐binding site.

### 2.4 Altered Labeling and Recovery of Prazole‐Tsg101 Adducts Identifies Further Structural Relationships

As our prior assay recovered structural information for only half the tested compounds, we sought to optimize our labeling and recovery of Prazole‐Tsg101 adduct spectra. Solubility was a major limitation for the bulky prazole derivatives, so we shifted to dilute labeling (1 µm Tsg101, 2.5x excess prazole) in a more physiological buffer (PBS) for 48 h, with the recovery of the Tsg101‐prazole adduct into NMR buffer. Adduct formation was assayed by both LC/MS (Table 1) and HSQC. Labeling in PBS slowed the attachment rate compared to the mildly acidic NMR buffer, but as indicated above may be reflective of the challenges faced by prazoles in Tsg101 adduct formation in the cell. In total, we trialed five commercial prazoles, the eighteen previously described derivatives, and a prazole dimer based on rabeprazole (compound 20).

**Figure 4:**
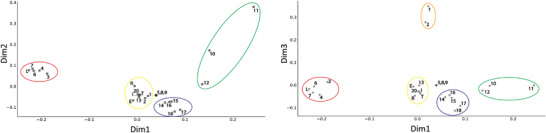
Global analysis of prazole‐adduct spectra identifies structurally distinct groups of bulky adducts and the formation of an anomalous adduct. Plots of the first two dimensions (top) and the first and third dimensions (bottom) from multiple correspondence analysis of the peak positions from 24 prazole‐adduct HSQC experiments. Spectra were collected to comparable SNR, after which they were peak‐picked and binarized, where peak centers were given a value of 1. The locations of the free shifts were added to all spectra to remove differences in labeling, and spectra were downsampled by a factor of 8, vectorized, and then used for MCA. Looking at 21 of the 24 tested compounds fall into five groups Figure 4, circled), while three (5, 8, 9) are identical and showed no evidence of attachment by NMR or LC/MS. The yellow group contains commercial prazoles and compounds 13 and 20 and was effectively unseparated, without major similarity in chemical shift. The orange group (1, 2) was distinct only in dimension 3 and showed highly similar shifts with multiple sets of peaks indicative of exchange or multiple adduct species. The group in blue contains derivatives having rabeprazole‐derived linkers. Their chemical shifts were similar and lacked extreme features, probably indicative of the flexibility afforded by the extended linker. The three compounds in green (10,11,12) showed a similar mode but with clearly separated shifts, where the dimension 1 and 3 values appear to correspond to the strength of the shift at several sites. Finally, the red group has negative values along the first dimension and is comprised of a mixture of sizes, linker lengths, and commercial prazole (lansoprazole). These spectra had nearly identical shifts and showed an anomalous, identical adduct mass by LC/MS, indicating the formation of a common secondary adduct on the UEV surface.

We initially attempted to perform PCA on the resulting set of 24 spectra, but the first three components continued to report primarily on the efficiency of adduct formation rather than on structural relationships (not shown). Instead, we opted to perform multiple correspondence analysis (MCA), a categorical counterpart to PCA, using discretized chemical shift data. The shift to MCA removed the impact of intensity, allowing us to focus on chemical shift similarity across compounds. Here, spectra were peak picked at a comparable signal‐to‐noise ratio (SNR) and binarized, where peak centers had a value of 1 and all other points were set to zero. The free shift positions were added to all spectra to remove the impact of variable labeling, after which spectra were downsampled 8x and unraveled into 1D vectors, which were then recombined into a 2D matrix for MCA (**Figure** **4**). This approach is similar to that used in the TREND NMR Pro software package for ligand screening and affinity determination, where we replaced the PCA analysis with binarization of the data and MCA to remove the impact of variable labeling across compounds.^[^ ^33^ ^]^

In the first three dimensions (axes), there are five apparent groupings (Figure 4, circled), while three (5, 8, 9) derivatives still showed no evidence of attachment by NMR or by LC/MS (Table 1). A group of commercial prazoles (except lansoprazole) and compounds 13 and 20 are circled in yellow. This cluster had little chemical shift similarity (Figure S3a, Supporting Information) and appeared poorly discriminated by MCA. The two smallest compounds (1, 2) are contained in this group for the second dimension but are separated into a distinct group in the third (orange). Their spectra overlay well (Figure S3b, Supporting Information) and clearly feature multiple sets of peaks. The next group (blue) comprises derivatives with rabeprazole linkers. Their spectra (Figure S3c, Supporting Information) are also well clustered, being most apparent for compounds 17 (purple) and 18 (orange). Compound 14 (red) is the most divergent, as expected given its phenyl rather than biphenyl R_3_. Next, both the first and third dimensions separate three bulky compounds (10, 11, 12) with the intermediate linker length, where in both 11 > 10 > 12. Their spectra (Figure S3d, Supporting Information) show this resulted from peaks where compound 11 (red) has the greatest shift, followed by 10 (blue) and then 11 (green).

### 2.5 One Group of Prazole Derivatives forms Irreducible Secondary Adducts on the Tsg101 UEV Domain

The Final group (red) has negative values in the first dimension, containing derivatives of mixed size, linker length, and commercial lansoprazole. Overlaying these spectra revealed, surprisingly, that they had major and minor sets of shifts, where the major set was identical (Figure S4, Supporting Information). This suggested a common pose or Tsg101‐prazole adduct, and so we looked at the LC/MS results. While most compounds had the expected adduct mass, compounds in this group displayed a high percentage of an anomalous species of +252 Da (Table 1). In addition to lansoprazole, the other compounds in this group (3, 4, 6, 7) shared the shortest R_3_ linker. Further comparison of LC/MS for compounds 6 and 12 (Figure S5, Supporting Information, left), which are identical excepting the longer linker in 12, showed the latter did not form the anomalous +252 Da species. We then checked for reversibility of the anomalous adduct using excess DTT (Figure S5, Supporting Information, right). For both lansoprazole and compound 6, DTT resulted in the loss of peaks associated with the expected adduct, but the +252 Da species proved irreversible. Contrastingly, compound 12, with only the expected adduct mass, was completely reversible with DTT treatment. The anomalous species thus represented an irreversible adduct, implying rearrangement from the initial disulfide attachment at C73 or secondary attack by a nearby residue to form an irreversible linkage.

**Figure 5:**
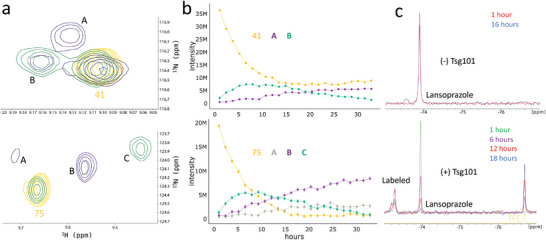
The secondary adduct follows initial adduct formation and involves the loss of the R3 group on the pyridine ring. A) Lansoprazole adduct peaks formed in the vicinity of sites 41 (top) and 75 (bottom) after 7 (green) and 29 h (purple), where the free peak prior to prazole addition is shown in yellow. Spectra several hours after prazole addition, when we find optimal viral inhibition, are a mixture of free Tsg101 UEV domain and an initial species, which gives rise to a secondary species marked by an additional confirmation or exchange. This is also seen by following the intensities of these species over time B), where the free peak is lost continuously, the initially formed species (peak B for site 41 and peak C for site 75) is lost over time, replaced by the secondary species (peak B for site 41 and peaks A and B for site 75). C) To test for the group lost during secondary adduct formation, we used ^19^F NMR with lansoprazole, which has a trifluoro group at the R3 position. With 100 µm lansoprazole in NMR buffer (top), there is minimal loss of the initial drug signal over 16 h (red to blue), where the slight shoulder may represent a degradation product. However, when stoichiometric Tsg101 UEV domain is present (right), there is instead a rise and fall of an apparent labeled species coupled with a steady loss of pro‐drug toward a new species which matches the chemical shift of trifluoroethanol (the R3 group of lansoprazole) collected under the same conditions (yellow). Error bars in (b) are derived from the noise level determined using NMRPipe and NMRDraw for each spectrum and are below the visible threshold for residue 41.

The +252 Da mass difference matched that of the core prazole minus R_3_, and we speculated that this group was lost before or after attachment to the UEV domain. To answer this question, we collected sequential HSQCs for the commercial lansoprazole, where peaks near two sites (41 and 75), at 7 (green) or 24 (purple) hours post‐addition are shown in **Figure** **5**a and their intensities over time are given in Figure 5b. In both cases, the drop in intensity of the free shift (yellow) is accompanied by the growth of an initial species, peaking at 5–10 h post‐addition, which then declines in favor of a second species. Notably, the trifluoro R_3_ group in lansoprazole allowed us to also follow this process by ^19^F NMR (Figure 5c). Without Tsg101, free lansoprazole shows a single primary peak over a 16‐h duration, with the appearance of a single side peak that may represent a minor degradation product. With Tsg101, however, the loss of the initial species is coupled to the putative prazole‐Tsg101 adduct species (at left). As in the HSQC series, this species peaks ≈6 h (purple) post‐addition before declining. Finally, there is a corresponding growth of a second species, which matches a trifluoroethanol (TFE) reference (yellow) prepared in an NMR buffer. Together, NMR and LC/MS suggest the formation of a secondary adduct on the surface of Tsg101, which follows the formation of the expected Tsg101‐prazole adduct. The secondary adduct is accompanied by loss of the R_3_ group and is seen for lansoprazole and derivatives with our shortest R_3_ linker. The adduct was also irreversible, suggesting it stems from the rearrangement of the initial disulfide linkage to a carbon‐sulfur bond, or forms from a secondary attack by a nucleophilic group on the protein such as the nearby K90 residue.

Given that the formation of the irreversible secondary adduct occurs in vitro in the presence of protein, the nucleophile may be a nearby protein sidechain, potentially contributed by lysine, arginine, or cysteine residues. In our previously determined pose for tenatoprazole on the surface of the Tsg101 UEV domain (PDB ID: 5VKG), there are three lysine residues (K33, K36, and K90) nearby to C73 (Figure S6, Supporting Information, blue), plus the second cysteine (C87). K33 is >15 Å from C73, while C87 is internally oriented and unlikely to participate. This left K36 or K90 as likely nucleophiles for secondary adduct formation. Approximating the available rotamers for both lysine residues (Figure S6, Supporting Information, blue spheres) shows that K90 is in near direct contact with the prazole, while approach of K36 is likely blocked by beta‐sheet sidechains, such as V38 and T56. As the irreversible adduct involves loss of the R3 group from the 4‐pyridyl position, we also modeled in rotation of the prazole about its final three chi angles, starting from the sulfur–sulfur bond, and plotted the resulting 4‐pyridyl positions (Figure S6, Supporting Information, gray spheres). The resulting distribution overlaps the K90 rotameric space but is >4 Å from the K36 space at all positions, indicating that K90 is the most likely contributor to secondary adduct formation.

The formation of irreversible adducts with lansoprazole and omeprazole has been previously observed for N‐terminal cysteine‐containing model peptides, albeit with a different observed delta mass (+233.1 Da) for the lansoprazole adduct,^[^ ^35^ ^]^ apparently reflective of the peptide nucleophile being the backbone amine of the N‐terminal Cys residue, rather than a lysine side chain. We thus wanted to test if irreversible adducts of our observed mass (+251–2 Da) could be produced in simple peptide systems having only cysteine and lysine sidechain nucleophiles. To do this, we tested several peptides of sequence GCG_n_K, where all peptides were N‐terminally acetylated to prevent the N‐terminal adduct seen by Watson et al. All tested peptides formed primary adduct with either stoichiometric or 3x excess addition of the commercial lansoprazole (Figure S7, Supporting Information), and a detectable secondary adduct of +251 Da. Significant quantities of the secondary adduct were detected only for the 4mer GCGK, indicating that favorable orientation and differences in pKa may contribute to the increased secondary adduct formation on the Tsg101 UEV surface. Still, Watson et al. speculated that irreversible adducts might be formed in the context of the parietal proton pump, and others have used resistance to treatment with reducing agents to identify supposed noncovalent interactions with prazoles,^[^ ^35^, ^36^ ^]^ which may then be complicated by the formation of irreversible covalent adducts. Consistent with these prior results, we also found secondary adducts formed with esomeprazole and with compound 13, where the latter lacked an R_2_ methyl and revealed through its simplified chemical shifts that the R_2_ methyl position likely forms substantial interactions with the UEV surface (Figure S4, Supporting Information).

Also surprisingly, the smallest derivatives (1, 2), displayed efficient labeling in the mildly acidic pH 5.8 NMR buffer, but minimal attachment (<20%) in the dilute PBS screen. We repeated the dilute labeling reaction with excess compound 1 and collected LC/MS directly for Tsg101 in both buffers, 24 h post‐addition (Figure S8a, Supporting Information), where mildly acidic conditions had > 90% adduct formation and both primary and secondary adducts. With PBS, however, labeling was significantly reduced (< 50%) with the expected and secondary adducts plus a mass consistent with the attachment of two prazoles, probably to both C73 and the nearby C87 in the Tsg101 UEV domain. This suggested some combination of slower prodrug conversion in the higher pH conditions and decreased stability of the small derivatives, which we followed by ^1^H NMR (Figure S8b, Supporting Information). In low and high pH conditions, compound 1 displayed a loss of the parent pro‐drug peaks, which was reduced at greater pH. However, there was no evidence of secondary peaks consistent with the activated species, whereas tenatoprazole (Figure S8c, Supporting Information) clearly formed a second species in both conditions. Additionally, overlaying an early HSQC of compound 1 at low pH showed minor peaks consistent with those of the adducts formed in PBS (Figure S4b, Supporting Information). This suggested that compounds 1 and 2 may be trapped in an intermediate conformation or state. Based on these results, we posit that the smallest phenyl derivatives, which rapidly form adducts in mildly acidic conditions, are limited by both prodrug conversion rate and stability under neutral conditions.

### 2.6 Many Tested Prazole Derivatives Display Significantly Improved Antiviral Activity over Commercial Prazoles and Are Effective Against SARS‐COV‐2

We next assayed most of the derivatives for efficacy in inhibiting HIV‐1 production, as we have done previously for several commercial prazoles,^[^ ^7^, ^8^ ^]^ noting that compounds 8, 9, and 13 were omitted due to solubility. Compounds were assayed in HEK293T cells for both the concentration at which VLP production was halved (EC_50_) and cytotoxicity (CC_50_) based on a WST‐1 metabolic assay, where reduction of the WST‐1 salt by NADH produces formazan dye proportional to the metabolic activity of the cell culture.^[^ ^37^ ^]^ Favorable targets should then display a high CC_50_/EC_50_ ratio (SI).^[^ ^8^ ^]^ The results are shown in **Table** **2**, where values of several commercial prazoles are reproduced from.^[^ ^7^ ^]^ We previously noted that efficacy correlated to conversion rate (by ^1^H NMR) for commercial prazoles, likely due to steric bulk or hydrophobicity.^[^ ^8^ ^]^

**Table 2:** Summary of the inhibition of HIV‐1 VLP production or SARS‐COV‐2 cytopathy by tested prazoles.

	EC_50_ HIV‐1 [µm]	CC_50_ HIV‐1 [µm]	SI HIV‐1	EC_50_ COV‐2 [µm]	CC_50_ COV‐2 [µm]	SI COV‐2
**Tenatoprazole**	50	125	2.5	54	100	1.9
**Lansoprazole**	25	50	2			
**Rabeprazole**	15	150	10			
**Ilaprazole**	22	42	1.9			
**Esomeprazole**	75	75	1			
**Compound 1**	No Effect	–	–			
**Compound 2**	No Effect	–	–			
**Compound 3**	3.5	37.5	10.7	6	36	6
**Compound 4**	1	6	6	2	50	25
**Compound 5**	6.5	30	4.6	50	50	1
**Compound 6**	3	95	31.7			
**Compound 7**	20	125	6.3			
**Compound 10**	0.5	10	20	2.5	50	20
**Compound 11**	2	5	2.5	2	75	37.5
**Compound 12**	20	80	4			
**Compound 14**	4	10	2.5	17	55	3.2
**Compound 15**	5	>100	>20			
**Compound 16**	3.5	110	31.4	6.5	50	7.7
**Compound 17**	5	>100	>20			
**Compound 18**	2	75	37.5	50	50	1
**Compound 19**	3	80	26.7			
**Compound 20**	1	1	1			

Most derivatives had improved EC_50_ values relative to the commercial prazoles, with only four (1, 2, 7, 22) having values >10 µM. In our previous characterization of the commercial prazoles, we observed a correlation of the conversion rate with the EC_50_ of viral inhibition. This was not observed for the present derivatives, implying most compounds crossed the effective concentration threshold within the 6‐h pretreatment time. Instead, the size of the R_3_ position appears to correlate with EC_50_, where the lowest values are derivatives with R_3_ biphenyls (4, 10, 11, 18) and the dimer (20). Apart from 18, these derivatives all display unfavorable SI ratios, implying surface bulk may also block an endogenous interaction. Increased flexibility via the rabeprazole linker (as in 14, 15, 16, 17, 18) greatly increases SI. These rabeprazole derivatives also clustered in the global analysis with similar shifts by 2D NMR, which were less extreme than those of compounds 10 and 11. Three other compounds had favorable EC_50_ and CC_50_ values (6, 7, 12). Derivatives 6 and 12 both have a biphenyl with a carboxylic substituent, differing in their linker length, while 7 replaces the carboxy with a tetrazole. Thus, all contain a biphenyl with a hydrophilic/acidic substituent. The preference for hydrophilic groups and flexibility in those derivatives having favorable SI ratios suggests that the prazole‐Tsg101 adduct may need to accommodate an additional interaction partner while blocking Ub. This may not necessarily involve the PTAP pocket, however, as in the intensity‐based analysis larger compounds tended to have reduced impact in this region.

The formation of the irreducible adduct was not correlated to EC_50_ or CC_50_ in HIV‐1 (compare compounds 3, 19, 23). This may be explained by the 6‐h preincubation time used in the VLP assays, which as shown in Figure 5 reflects the maxima for the expected adduct before significant formation of the secondary adduct. Still, the ability to form a nonreducible adduct on the cell surface is likely to be an attractive avenue both to make prazoles more specific to Tsg101, to increase their half‐life in the cell, and to give more design freedom for optimizing membrane passage, knowing that groups at the R_3_ position can be removed to form the final active species. Similarly, timing may explain the lack of efficacy seen for compounds 1 and 2, which appeared to be both slowed and unstable in more physiological conditions.

In addition to HIV‐1, a subset of derivatives was also tested in CALU3 cells against SARS‐COV‐2 (Table 2, right), which has recently been suggested as an attractive target for prazole inhibition.^[^ ^38^ ^]^ Although a recent meta‐analysis of commercial prazoles with SARS‐COV‐2 has found paradoxical or limited association.^[^ ^39^, ^40^ ^]^ Prazoles appear effective at inhibiting SARS‐COV‐2 production, where most tested derivatives had EC_50_ > the commercial tenatoprazole. Three (5, 14, 18) had significantly worse EC_50_ values than those seen for HIV‐1 VLP production, whereas the remainder (3, 4, 10, 11, 16) were comparable. Interestingly, the apparent benefit of increased flexibility at the R_3_ position seen in HIV‐1, was not seen for SARS‐COV‐2, where two of the derivatives with rabeprazole linkers (14, 18) had significantly reduced efficacy and no strong deviation was seen in the CC_50_ values for the tested derivatives. The results thus suggest that SARS‐COV‐2 is a viable target for prazole‐based treatment but tailored development may be required between virus families.

### 2.7 Reduced Sulfide Derivatives Are Unable to Label Tsg101 UEV In Vitro but Are Comparably Effective to the Parent Prazole‐Sulfoxide in Cellular Assays

In addition to looking downstream at the formation of secondary species after the initial prazole‐Tsg101 adduct, we also looked upstream at the viability of a sulfide derivative, which must be converted *in‐cell* to the sulfoxide pro‐drug. This compound (19) was derived from our fastest‐attaching sulfoxide (14) and displayed no evidence of Tsg101 interaction or attachment in vitro by NMR (Figure S9, Supporting Information). The intensity reduction at C73 was only a few percent, while the parent sulfoxide (14) had almost complete intensity loss (Figure S9a, Supporting Information) and the sulfide produced no apparent chemical shifts (Figure S9b, Supporting Information). The sulfide was equivalent to the parent sulfoxide, however, in EC_50_ and CC_50_ in the HIV‐1 VLP assay (Table 2), indicating that it was effective within the cell. We showed previously that N‐acetyl cysteine (NAC) can compete with tenatoprazole for cysteines, such as Tsg101 C73, where the presumptive reduction in prazole‐Tsg101 adduct alleviated suppression of viral particle formation.^[^ ^7^ ^]^ Figure S10 (Supporting Information) shows that NAC is similarly able to compete with the sulfide derivative, suggesting that it also forms a disulfide adduct and thus was putatively converted to the parent compound. Prazoles are known to be degraded in the liver primarily by members of the CYP450 family, principally through the formation of hydroxyl, sulfide, or sulfone derivatives.^[^ ^26^ ^]^ The latter, the sulfoxidation process, which ordinarily would oxidize the sulfoxide to the inactive sulfone, is likely responsible for the activation of the sulfide to the sulfoxide pro‐drug. Further work will be required to determine the enzyme responsible for this activation, although the ability to employ sulfide derivatives opens another design avenue, as pro‐drug activation can be delayed until after membrane passage into the cellular interior, blocking extracellular conversion that is non‐viable in the antiviral context.

## 3 Conclusion

Here, we present in vitro characterization of Tsg101 adduct formation for a family of prazole derivatives together with the characterization of their ability to inhibit viral particle production by HIV‐1 and SARS‐COV‐2, where we identify the latter as a novel target for prazole inhibition. Screening of Tsg101‐prazole adduct formation by solution NMR revealed that larger compounds tended toward increased impacts at the Ub‐binding site instead of the PTAP pocket. This was confirmed by subsequent global analysis, which also revealed a subset of compounds that form the same irreversible secondary adduct on the UEV surface, which could be leveraged for increased therapeutic half‐life or for the design of sacrificial R_3_ substituents for membrane passage or reduction of off‐target adduct formation. Suppression of HIV‐1 VLP production was tied to increased size at the R_3_ position where cytotoxicity was improved by increased flexibility in the R_3_ linker. Finally, we determined that sulfide derivatives are effective at blocking viral particle production, putatively through cellular sulfoxidation, allowing further targeting through delayed conversion. Together, these results open multiple avenues for the design of prazole derivatives for antiviral applications.

## 4 Experimental Section

### In Vitro Characterization of Prazole Derivatives—Production of Tsg101 UEV Domain

Tsg101 UEV domain (residues 2–145), together with an N‐terminal His_6_ tag in a pET‐28B‐vector was transformed into Rosetta 2 (DE3) pLysS cells, grown in M9 medium supplemented with ^15^NH_4_Cl, and induced with IPTG. Cell pellets were solubilized in lysis buffer (100 mm Tris, 100 mm NaCl, 10% glycerol, pH 7.5) plus a protease inhibitor cocktail tablet (Roche) and benzonase nuclease (Millipore). Cells were lysed with an emulsifier (Emulsiflex‐C3) and pelleted by ultracentrifugation (40K rpm, 60 min, 4 °C, Beckman).

The supernatant was run over a nickel affinity column (HisTrap FF, GE Healthcare), with gradient elution to 500 mm imidazole. His tag was cleaved with TEV protease overnight at room temperature, after which cleavage was checked by sodium dodecyl sulfate (SDS)‐PAGE electrophoresis, and excess imidazole was removed by concentration in a 3K cutoff spin‐concentrator (Pall) back into the lysis buffer. The protein was run back through a second HisTrap FF to remove uncleaved protein and then passed onto a size‐exclusion column (HiLoad 16/60 Superdex 75 pg, GE Healthcare), equilibrated with NMR buffer (20 mm potassium phosphate, 50 mm NaCl, pH 5.8). The Tsg101 fractions were pooled and concentrated, where concentration was estimated by A280 on an ND‐1000 spectrophotometer (Thermo Fisher) and purity was assessed by SDS‐PAGE, and the presence of Tsg101 UEV was confirmed by LC/MS (Agilent 6224 ESI‐ TOF).

### In Vitro Characterization of Prazole Derivatives—NMR Spectroscopy

1D NMR data were acquired at 300 K on a Bruker 600 MHz spectrometer equipped with a room temperature probe. ^1^H NMR was recorded with 1024 scan, 30 min intervals to follow compound 1 and tenatoprazole conversion for 1 mm compound in 20% DMSO (70 µL) and 80% NMR buffer, or 80% NMR buffer adjusted to pH 7.4, where the NMR buffer was prepared with 10% D_2_O. ^19^F NMR spectra to monitor lansoprazole attachment and R3 liberation were recorded with 1536 scan (71 min) intervals with either 100 µm lansoprazole pro‐drug in NMR buffer, or the same with the addition of 100 µm Tsg101 UEV, both with 10% D_2_O. The liberated R3 group was matched to a sample of TFE in NMR buffer at 1 mm recorded under the same conditions. ^19^F shifts were referenced to 10% TFA, aqueous (−75.5 ppm). 2D NMR data were recorded on an 800 MHz spectrometer equipped with a cryogenic probe. All spectra were recorded at 300 K and initially processed using NMRPipe^[^ ^41^ ^]^ and viewed using CCPNMR 3.04^[^ ^42^ ^]^. Further processing used Python with the nmrglue, pandas, and plotly packages for visualization.

### In Vitro Characterization of Prazole Derivatives—Time‐Dependence of Prazole Attachment by Solution NNMR

To follow the time‐dependence of prazole attachment to the Tsg101 UEV domain, samples containing 300 µL of 100 µm of ^15^N Tsg101 UEV in NMR buffer were combined with 20 µL of D_2_O (Cambridge Isotope Laboratories) to a final volume of 320 µL and transferred to a Shigemi tube. A free control spectrum was recorded with 16 scans (duration 83 min), after which stoichiometric prazole (in dimethylsulfoxide solvent) was added to the tube, where the stock concentration was adjusted to ensure a volume addition of 2–2.5 µL DMSO. The same HSQC settings were then used to record 25 subsequent experiments at the same 83‐min intervals, and the process was repeated for each compound. All spectra were analyzed using the same NMRPipe scripts, except for individual phase correction.

For intensity‐based analysis, peaks were picked at equivalent contour levels in CCPNMR 3.04 and the intensities of all free peaks at the 5th point post‐prazole addition (≈7 h) were determined. These values were normalized relative to the intensities of the free control collected prior to prazole addition and plotted against the UEV domain residues for each compound. For the site of covalent attachment (C73) specifically, the intensity of the free C73 peak was extracted for all tested spectra, again normalizing to the height of the free controls, and fit the resulting curves to a simple exponential decay function: *I_t_* = *e* ^−*kt*^  + *I_f_* to estimate the degree of labeling and the rate of attachment to C73. Intensity processing used python and the nmrglue, pandas, and scipy packages. Decays were fit using the curve fit function and the above decay function, where the sigma of the data was taken to be the noise level estimated using NMRDraw for each starting spectrum, and standard deviations of the fit parameters were calculated from the resulting covariance matrix.

Global analysis of the spectra used the principal component analysis module integrated into NMRPipe, which was accessed via the specView.tcl script. Prior to PCA analysis, spectra were corrected with a polynomial baseline fit in NMRPipe and intensity‐normalized to the most intense peak. The various regions of the spectra were tried by PCA, where the best results were found for the spectral region from 7.5 to 9.4 ppm in the ^1^H dimension, and 112 to 130 ppm in the ^15^N dimension, which corresponded to the densest, central region of the HSQCs. The use of the full spectrum produced the same trends, but with reduced separation.

### In Vitro Characterization of Prazole Derivatives—Characterization of Prazole‐UEV Adducts by Solution NMR

To gain further structural information on the commercial prazoles and tested prazole derivatives, to shift from kinetics to focusing on the endpoint adduct opted. To do this, dilute labeling of all tested derivatives with the Tsg101 UEV domain was performed, where the UEV was kept at a concentration of 1 µm in phosphate‐buffered saline (CrystalGen) for 48 h in the presence of 2.5‐fold excess prazole. Samples were kept at RT in foil with mild shaking (50 rpm) for the duration. Labeling was assayed by LC/MS and samples were transferred to NMR buffer by spin‐concentration, where buffer exchange was monitored by pH of the flow through. Recovered protein concentration was estimated by A280 and used to adjust the number of scans (relative to a base *n* = 16) in the subsequent HSQC experiments to obtain comparable SNR.

For global analysis of the resulting spectra by multiple correspondence analysis, spectra were first analyzed in NMRPipe as before. Processed spectra were then peak‐picked in CCPNMR 3.04 at a threshold value just above the noise, adjusted for SNR by the square root of the number of scans, and picked peaks were given ambiguous identifiers. Peak positions were recorded, and the data were binarized, where points having a peak center were set to a value of 1 and all other points were given a value of zero. The spectral region from 5.4 to 10.5 (1H) and 104 to 132 (15N) ppm was then extracted, corresponding to a 512 by 768 data matrix. To account for incomplete labeling, the positions of the free Tsg101 UEV peaks were set to one in all spectra, effectively removing the contribution of these locations from the subsequent analysis. Spectra were then downsampled by a factor of eight in both dimensions, to reduce imprecision and the impact of small shifts. The downsampled spectra were then flattened to 1d arrays and the prince factor analysis package in Python was used for the MCA, where the first three dimensions are looked at. The resulting dimension plots were then used to identify similar structures, where groups were determined visually and by shared chemical or structural features.

### In Vitro Characterization of Prazole Derivatives—*LC/MS of Tsg101‐Prazole Adducts*

LC/MS data were collected on Agilent 6530 or G1956B instruments for the dilute labeling screen described above where LC/MS was run on the recovered adduct, and also to test for the irreducibility of the secondary adduct and to assess the labeling of compound1. For the irreducibility screen, 10 µm Tsg101 UEV in NMR buffer was combined with a twofold excess of compounds 6, 12, or lansoprazole and incubated for 24 h at RT. Half of the labeled sample was then removed, diluted with pH 8 buffer to adjust the final buffer pH to neutral, and 10 mm DTT was added for 30 min to assay the reducibility of the prazole‐adduct species. For the compound 1 screen, 10 µm Tsg101 UEV in NMR buffer or PBS was combined with a twofold excess of compound one and again incubated for 24 h at RT. Mass and UV spectra were analyzed and deconvoluted using the software MassHunter Qualitative Analysis version B.07 (Agilent Technologies, CA, USA) with BioConfirm.

### In Vitro Characterization of Prazole Derivatives—*LC/MS for CK Peptide‐Prazole Adducts*

Peptides of general sequence GCG_n_K were obtained from Genscript, and were N‐terminally acetylated and C‐terminally amidated. Peptides at 100 µm concentration were incubated in the pH 5.8 NMR buffer with 1x or 3x lansoprazole for 24 h, after which the product was collected for LC/MS. HPLC‐mass spectrometry was analyzed on an Agilent 6530C quadrupole‐time of flight (QTOF) mass spectrometer with an Agilent 1200 series capillary system equipped with a reverse‐phase column (ZORBAX 300SB‐C18, 2.1×50 mm, 3.5 mm, Agilent Technologies, DE, USA) set to 30 °C. The analyte was followed using not only the accurate mass but also the UV chromatogram at 281 nm. Mass and UV spectra were analyzed and deconvoluted using the software MassHunter Qualitative Analysis version B.07 (Agilent Technologies, CA, USA) with BioConfirm.

### Statistical Analysis

All NMR data were processed using standard processing protocol. Error estimates were determined by Monte Carlo using the estimated spectral noise added to the fitted data.

## Conflict of Interest

The authors declare no conflict of interest.

## Supporting information

Supporting Information

## Data Availability

The data that support the findings of this study are available in the supplementary material of this article.
