# Supplementary material for: Structural Relationships to Efficacy for Prazole‐Derived Antivirals
Source: Adv Sci (Weinh). 2024 Mar 6;11(18):2308312. doi: 10.1002/advs.202308312 (PMC11095225; doi:10.1002/advs.202308312)
Supplement: Supplementary file 1 — Supporting Information [file ADVS-11-2308312-s001.pdf]

ADVANCED SCIENCE
Open Access

Supporting Information

for *Adv. Sci.*, DOI 10.1002/advs.202308312

Structural Relationships to Efficacy for Prazole-Derived Antivirals

David A. Nyenhuis, Susan Watanabe, Rebecca Bernstein, Rolf E. Swenson, Natarajan Raju, Venkata R. Sabbasani, Chandrasekhar Mushti, Duck-Yeon Lee, Carol Carter and Nico Tjandra**

Supporting Information
©Wiley-VCH 2023
69451 Weinheim, Germany

Structural Relationships to Efficacy for Prazole-Derived Antivirals

David A. Nyenhuis[a], Susan Watanabe[b], Rebecca Bernstein[a], Rolf E. Swenson[c], Natarajan Raju[c], Venkata R. Sabbasani[c], Chandrasekhar Mushti[c], Duck-Yeon Lee[d], Carol Carter[b], Nico Tjandra[a*]

Table of Contents

Experimental Procedures 3
Reagents 3
Synthesis of Prazole Derivatives 3
Synthesis of Compounds 1-3 3
Synthesis of Compounds 4-19 5
    Scheme I 5
    Scheme II 6
    Scheme III 7
    Detailed Preparation of Compounds 4-19 7
Synthesis of Compound 20 8
Prazole Derivatives 10
SARS-COV-2 Screen 17
HIV-1 VLP Assays 17
Results 18
    Figure S1 18
    Figure S2 19
    Figure S3 20
    Figure S4 21
    Figure S5 22
    Figure S6 23
    Figure S7 24
    Figure S8 25
    Figure S9 26
    Figure S10 27
References 28
Author Contributions 28

Experimental

Reagents

Commercially Available Prazoles and Reagents for Synthesis

Tenatoprazole (Selleckchem, S4212), lansoprazole (Selleckchem, S1354), esomeprazole (Sigma, E7906), rabeprazole (Selleckchem, S4845), and ilaprazole (Selleckchem, S3666) were obtained commercially. Reagents for synthesis were procured from Sigma Aldrich, Oakwood products, and Ambeed Chemicals, Inc. Solvents were purchased and used as received from Sigma.

Instrumentation used in Synthesis of Prazole Derivatives

NMR spectra for verification were recorded on Bruker-400 and Varian 400 MHz instruments. LC/MS were run and recorded on an Agilent 1200 LC/MS instrument.

Conditions used for Preparative and Analytical HPLC

Preparative HPLC conditions: Column 30x250 mm Waters X-Terra C18 RP column with a particle size of 5µ, and with 130°A pore size. Water (solvent A with 0.1% NH_4OH, v/v), and Methanol (B, 0.1% NH_4OH, v/v) were used as eluents. Gradient: 10-100% over 40 min with a flow rate of 30.0 ml/min. Detection @220 nm and 254 nm.
Analytical HPLC Conditions: (A) Column Zorbax (Agilent) C18 RP, 120°A; 5.0 micron; 4.6 x 50.0 mm; solvent A – Water; Solvent B- methanol with 5mM ammonium acetate/acetic acid (pH~ 6.0).; Elution rate; 1.0 ml/min; Gradient 5-100%B over 15.0 min. (B): Column Porosil 120°A; 2.7 micron; 4.6 x 50.0 mm; solvent A – Water; Solvent B- methanol with 5mM ammonium acetate/acetic acid (pH~ 6.0).; Elution rate; 1.0 ml/min; Gradient 5-95%B over 7.0 min

Synthesis of prazole derivatives

General Synthesis of Compounds 1-3

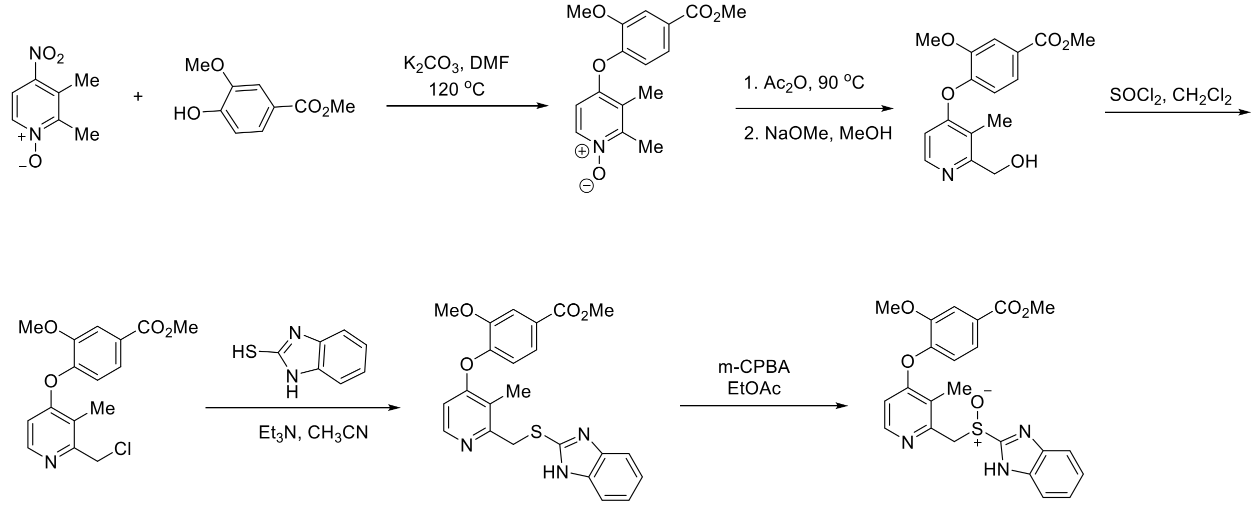

Synthesis of methyl 4-((2-(((1H-benzo[d]imidazol-2-yl)sulfinyl)methyl)-3-methylpyridin-4-yl)oxy)-3-methoxybenzoate (Compound 3): In a thick-walled flask, 2,3-dimethyl-4-nitropyridine 1-oxide (1 g, 5.95 mmol) and methyl 4-hydroxy-3-methoxybenzoate (1.27 g, 7.14 mmol) were dissolved in DMF (15 mL) and then K_2CO_3 (2.07 g, 15 mmol) was added at room temperature. The flask was sealed and heated at 120 °C for 15 hours. The reaction mixture was cooled to room temperature, water (50 mL) was added and extracted with CH_2Cl_2 (3 x 50 mL). The combined organic layers were washed with saturated aqueous NaCl (2 x 50 mL), dried over anhydrous Na_2SO_4, and filtered. The filtrate was concentrated and purified by ISCO combi flash silica gel column chromatography to afford 4-(2-methoxy-4-(methoxycarbonyl)phenoxy)-2,3-dimethylpyridine 1-oxide (199 mg, 11%).
^{1}H NMR (400 MHz, cdcl$_3$) δ 8.01 (dt, *J* = 7.2, 0.6 Hz, 1H), 7.68 – 7.61 (m, 2H), 7.02 – 6.95 (m, 1H), 6.40 – 6.33 (m, 1H), 3.90 (s, 3H), 3.82 (s, 3H), 2.54 (q, *J* = 0.5 Hz, 3H), 2.33 – 2.28 (m, 3H).
^{13}C NMR (101 MHz, cdcl$_3$) δ 166.16, 153.52, 150.72, 149.79, 147.01, 137.41, 127.95, 124.50, 123.16, 120.68, 113.86, 110.02, 56.00, 52.35, 14.46, 12.36. LC-MS $[M+H]^+$: 304.20 (m/z).

4-(2-methoxy-4-(methoxycarbonyl)phenoxy)-2,3-dimethylpyridine 1-oxide (3.4 g, 11.2 mmol) in acetic anhydride (22 mL) was heated at 90 °C for 8 hours. Acetic anhydride was removed by rotary-evaporator and the crude material was dried to use for the next step. The dried crude material was dissolved in MeOH (30 ML) and then NaOMe (3.66 M in MeOH, 15 mL, 56 mmol) was added dropwise at room temperature under inert atmosphere. The reaction mixture was stirred for 3 hours and then quenched with aqueous NH_4Cl (20 mL). Extracted with CH_2Cl_2 (3x50 mL), and the combined organic layers were dried over anhydrous Na_2SO_4. The filtrate was concentrated to afford crude methyl 4-((2-(hydroxymethyl)-3-methylpyridin-4-yl)oxy)-3-methoxybenzoate, which was used for the next step without further purification. To the solution of methyl 4-((2-(hydroxymethyl)-3-methylpyridin-4-yl)oxy)-3-methoxybenzoate (2.25 g, 7.42 mmol) in CH_2Cl_2 (20 mL), thionyl chloride (1.6 mL, 22.2 mmol) was added at room temperature. The reaction monitored by LC-MS for the consumption of starting material. After the disappearance of starting material, excess thionyl chloride was removed by rotary-evaporator and the crude material (methyl 4-((2-(chloromethyl)-3-methylpyridin-4-yl)oxy)-3-methoxybenzoate) was used for the next step. The crude product was dissolved in CH_3CN (20 mL) and then 1H-benzo[d]imidazole-2-thiol (1.11 g, 7.42 mmol) and Et_3N (2.1 mL, 14.8 mmol) at room temperature. The reaction monitored by TLC and LC-MS. After the consumption of starting materials, the solvent and excess triethyl amine were removed and the crude product was purified by ISCO combi flash silica gel column chromatography

(EtOAc/MeOH) to afford the methyl 4-((2-(((1H-benzo[d]imidazol-2-yl)thio)methyl)-3-methylpyridin-4-yl)oxy)-3-methoxybenzoate. LC-MS $[M+H]^+$: 436.00 (m/z).

To the solution of 4-((2-(((1H-benzo[d]imidazol-2-yl)thio)methyl)-3-methylpyridin-4-yl)oxy)-3-methoxybenzoate (3 g, 6.9 mmol) in EtOAc (60 mL), 3-chloroperbenzoic acid (1.54 g, 6.9 mmol) at -5 °C under inert atmosphere. The reaction was monitored by LC-MS for the disappearance of starting material (about 2 hours). The white precipitate was formed, which was collected and washed with cold EtOAc (3 x 20 mL) and then dried to afford methyl 4-((2-(((1H-benzo[d]imidazol-2-yl)sulfinyl)methyl)-3-methylpyridin-4-yl)oxy)-3-methoxybenzoate (1.98 g, 64%).

^{1}H NMR (400 MHz, dmso) δ 13.58 (s, 1H), 8.15 (dd, *J* = 5.6, 0.7 Hz, 1H), 7.78 – 7.49 (m, 4H), 7.29 (s, 2H), 7.20 (d, *J* = 8.3 Hz, 1H), 6.44 (d, *J* = 5.6 Hz, 1H), 4.98 – 4.68 (m, 2H), 3.86 (s, 3H), 3.80 (s, 3H), 2.28 (s, 3H).

^{13}C NMR (101 MHz, dmso) δ 166.01, 162.36, 154.65, 152.17, 151.39, 148.49, 146.59, 128.21, 123.29, 123.06, 122.38, 120.23, 114.18, 112.86, 109.48, 60.51, 56.42, 52.81, 11.36.

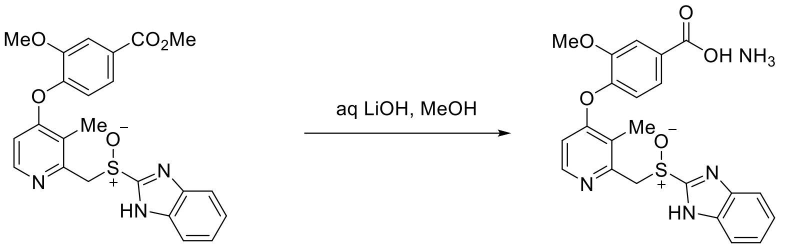

Synthesis of 4-((2-(((1H-benzo[d]imidazol-2-yl)sulfinyl)methyl)-3-methylpyridin-4-yl)oxy)-3-methoxybenzoic acid, ammonia salt: To the solution of 4-((2-(((1H-benzo[d]imidazol-2-yl)sulfinyl)methyl)-3-methylpyridin-4-yl)oxy)-3-methoxybenzoate (10 mg, 0.02 mmol) in MeOH (2 mL), aqueous LiOH (24 mg, 0.06 mmol) in water (1 mL) was added. After complete hydrolysis (monitored by LC-MS), the reaction diluted with water and adjusted the pH to 7 to 8 by slowly adding 1% acetic acid. The neutralized compound was purified by HPLC using water and acetonitrile (water was buffered with NH_4OAC (10 mmol) and adjusted the pH between 7 to 8 by adding 1 or 2 drops NH_4OH) to provide 4-((2-(((1H-benzo[d]imidazol-2-yl)sulfinyl)methyl)-3-methylpyridin-4-yl)oxy)-3-methoxybenzoic acid, ammonia salt (6 mg, 60%).

^{1}H NMR (400 MHz, d_2o) δ 7.90 (d, *J* = 5.8 Hz, 1H), 7.59 (dd, *J* = 6.2, 3.2 Hz, 2H), 7.53 (d, *J* = 1.9 Hz, 1H), 7.39 (dd, *J* = 8.4, 1.9 Hz, 1H), 7.29 (dd, *J* = 6.2, 3.2 Hz, 2H), 6.76 (d, *J* = 8.2 Hz, 1H), 6.44 (d, *J* = 5.7 Hz, 1H), 4.72 (s, 2H), 3.69 (s, 3H), 1.97 (s, 3H).

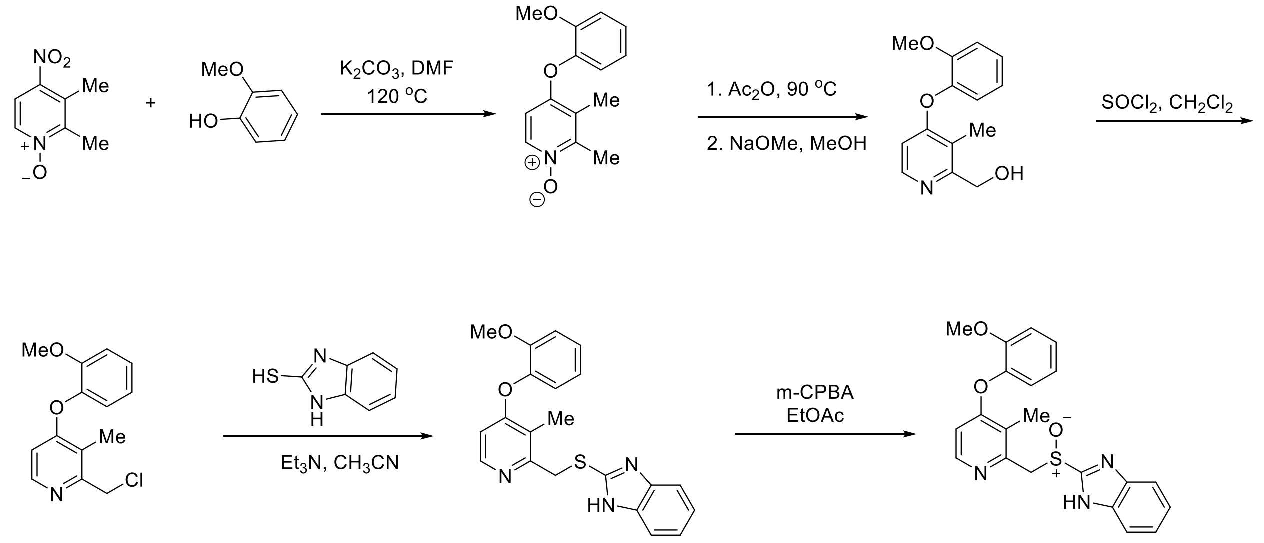

Synthesis of 2-(((4-(2-methoxyphenoxy)-3-methylpyridin-2-yl)methyl)sulfinyl)-1H-benzo[d]imidazole: 2-(((4-(2-methoxyphenoxy)-3-methylpyridin-2-yl)methyl)sulfinyl)-1H-benzo[d]imidazole was synthesized similar to that of methyl 4-((2-(((1H-benzo[d]imidazol-2-yl)sulfinyl)methyl)-3-methylpyridin-4-yl)oxy)-3-methoxybenzoate. The precipitate was not observed during the oxidation with m-CPBA and crude product was purified by ISCO combi flash basic alumina column chromatography (CH_2Cl_2/MeOH) to afford 2-(((4-(2-methoxyphenoxy)-3-methylpyridin-2-yl)methyl)sulfinyl)-1H-benzo[d]imidazole.

^{1}H NMR (400 MHz, $cdcl_3$) δ 8.20 (dd, *J* = 5.7, 0.6 Hz, 1H), 7.83 – 7.48 (broad peak, 2H), 7.37 – 7.18 (m, 3H), 7.07 – 6.92 (m, 3H), 6.38 (d, *J* = 5.6 Hz, 1H), 4.95 – 4.75 (m, 2H), 3.75 (s, 3H), 2.35 (s, 3H).

^{13}C NMR (101 MHz, $cdcl_3$) δ 163.72, 153.31, 151.54, 150.31, 147.90, 147.86, 142.45, 126.67, 122.86, 122.61, 121.35, 113.01, 108.88, 108.77, 60.92, 55.75, 11.31. LC-MS $[M+H]^+$: 394.20 (m/z).

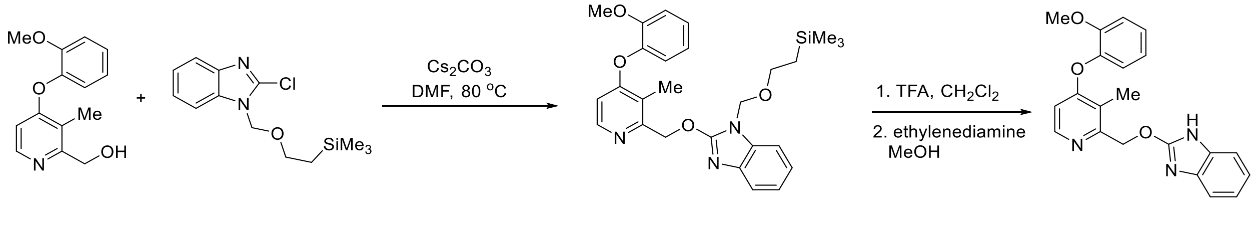

(4-(2-Methoxyphenoxy)-3-methylpyridin-2-yl)methanol (87 mg, 0.35 mmol) and 2-chloro-1-((2-(trimethylsilyl)ethoxy)methyl)-1H-benzo[d]imidazole (100 mg, 0.35 mmol) were dissolved in DMF (3 mL) and then Cs_2CO_3 (231 mg, 0.7 mmol) was added. The reaction mixture heated at 80 ^oC for overnight. Water (10 mL) was added and extracted with EtOAC (30 mL). The organic phase was washed with saturated aqueous NaCl (10 mL), dried over anhydrous Na_2SO_4, and filtered. The filtrate was concentrated and purified by ISCO combi flash silica gel column chromatography to provide 2-((4-(2-methoxyphenoxy)-3-methylpyridin-2-yl)methoxy)-1-((2-(trimethylsilyl)ethoxy)methyl)-1H-benzo[d]imidazole (45 mg, 26%). ^1H NMR (400 MHz, cdcl$_3$) δ 8.23 (dd, *J* = 5.6, 0.7 Hz, 1H), 7.72 – 7.52 (m, 1H), 7.38 – 7.32 (m, 1H), 7.25 – 7.14 (m, 3H), 7.09 – 6.97 (m, 3H), 6.43 (dd, *J* = 5.6, 0.5 Hz, 1H), 5.77 (s, 2H), 5.41 (s, 2H), 3.79 (s, 3H), 3.61 – 3.49 (m, 2H), 2.47 (t, *J* = 0.5 Hz, 3H), 1.02 – 0.74 (m, 2H), -0.07 (s, 9H). ^{13}C NMR (101 MHz, cdcl$_3$) δ 163.54, 157.07, 154.15, 151.64, 147.74, 142.63, 140.08, 133.67, 126.60, 122.59, 122.29, 122.14, 121.46, 121.35, 117.87, 113.04, 109.18, 109.12, 71.57, 71.32, 66.27, 55.87, 55.80, 17.78, -1.48.

2-((4-(2-methoxyphenoxy)-3-methylpyridin-2-yl)methoxy)-1-((2- (trimethylsilyl)ethoxy)me -thyl)-1H-benzo[d]imidazole (20 mg, 0.04 mmol) was dissolved in 1:1 mixture of CH_2Cl_2/TFA (0.4 mL) and stirred for 2 hours. The solvent and TFA were removed and the crude material was dissolved in MeOH (3 mL) and few drops of ethylenediamine was added. The mixture was stirred for 30 minutes. Solvent was removed and the crude material was purified by ISCO combi flash silica gel column chromatography to provide 2-((4-(2-methoxyphenoxy)-3-methylpyridin-2-yl)methoxy)-1H-benzo[d]imidazole (6 mg, 41%).

^{1}H NMR (400 MHz, cdcl$_3$) δ 8.27 (s, 1H), 7.54 – 6.92 (m, 9H), 6.45 (s, 1H), 5.78 (d, *J* = 3.7 Hz, 2H), 3.80 (s, 3H), 2.48 (s, 2H).

^{13}C NMR (101 MHz, cdcl$_3$) δ 163.81, 157.99, 154.03, 151.56, 147.38, 142.44, 126.73, 122.63, 121.62, 121.41, 121.38, 113.04, 109.08, 69.84, 55.77, 10.47. LC-MS $[M+H]^+$: 362.20 (m/z)

Synthesis of Compounds 4-19

Compounds used in this study were synthesized as detailed in the following schemes I-III. The initial step involved, either the formation of the aryl ethers (Scheme I) starting from the known alcohol 11 or alkylation of the commercially available 4-chloro-2,3-dimethylpyridine-1-oxide with the corresponding phenol/alcohol under basic conditions. Low yields were observed for 10a-c (5%-10%), though moderate yields were obtained (40-50%) for compounds 3a-c under Mitsunobu conditions. Compounds 10a-c were subjected to Suzuki coupling under standard conditions to yield the coupled biaryls 11a-h2 in respectable yields (50-60%). Rearrangement of the alkylated/coupled products from Scheme I and II by heating with acetic anhydride provided the pyridyl 2-acetoxymethyl derivatives that were taken to next step without further purification. Stirring the crude acetates with potassium carbonate in methanol provided the respective alcohols. It was determined that purification was not necessary at this stage, and the crude alcohols from 4a-c, and 12a-h were converted to the chloromethyl derivatives with thionyl chloride. The chlorides/hydrochlorides from the above step were pure enough for further alkylation with 2-thiobenzimidazole. Alkylation of the chloro-derivatives with 2-thiobenzimidazole in DMF, and in the presence of sodium bicarbonate yielded the precursors to the final sulfoxides. Purification was carried out at this stage by silica column chromatography using ethyl acetate and chloroform as eluents. Overall yields of 45-50% were observed for 4 steps (7a-c and 14a-h). Oxidation of the sulfides 7a-c, and 14a-h, yielded the sulfoxides with m-CPBA in ethyl acetate. Purification of final sulfoxides was carried out by preparative HPLC under mildly basic conditions to yield the sulfoxides 8a-c and 15 a-h (10-20%). Hydrolysis of the methyl esters containing the sulfoxide with lithium hydroxide in dioxane followed by preparative HPLC resulted in the sulfoxide-acids 9a,9b,16a, and 16c (25-30%).

Tetrazoles reported here were prepared as described in Scheme III. Conversion of the cyano to the tetrazole moiety was achieved by heating the cyano precursors (7c, 14b and 14d) with pyridine-hydrochloride (10.0 equiv.), and large excess of sodium azide (20.0 equiv.) in DMF to 70oC for 20h followed by silica gel column purification (chloroform/methanol with 0.1% ammonium hydroxide as eluents; 20%-30% yields, 17a-c). Oxidation of the sulfides to sulfoxides was effected with m-CPBA in ethyl acetate at 0oC for 30 min, although low yields were observed after preparative HPLC purification under basic pH of 9.0 (10-24%, 18a-c)

Scheme I

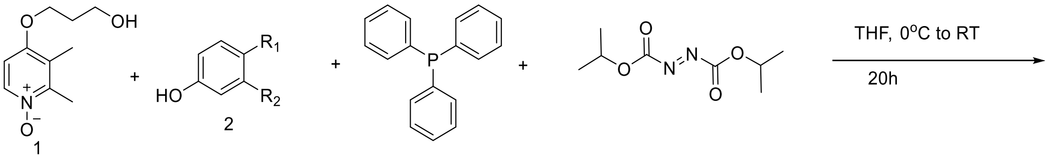

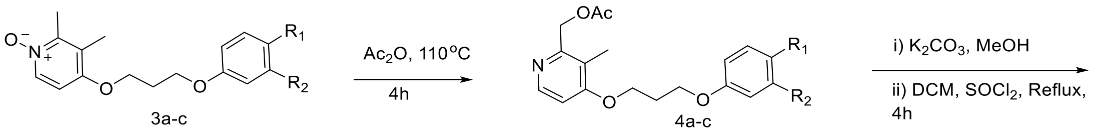

3a R_1=-COOMe; R_2 = -OMe; 3b R_1 = -3-Carbomethoxyphenyl; 3c = 3-Cyanophenyl; R_2 =H

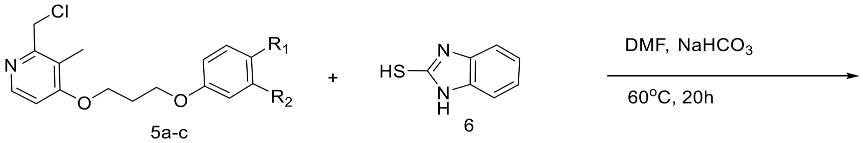

5a R_1=-COOMe; R_2 = -OMe; 5b R_1 = -3-Carbomethoxyphenyl; 5c = 3-Cyanophenyl; R_2 =H

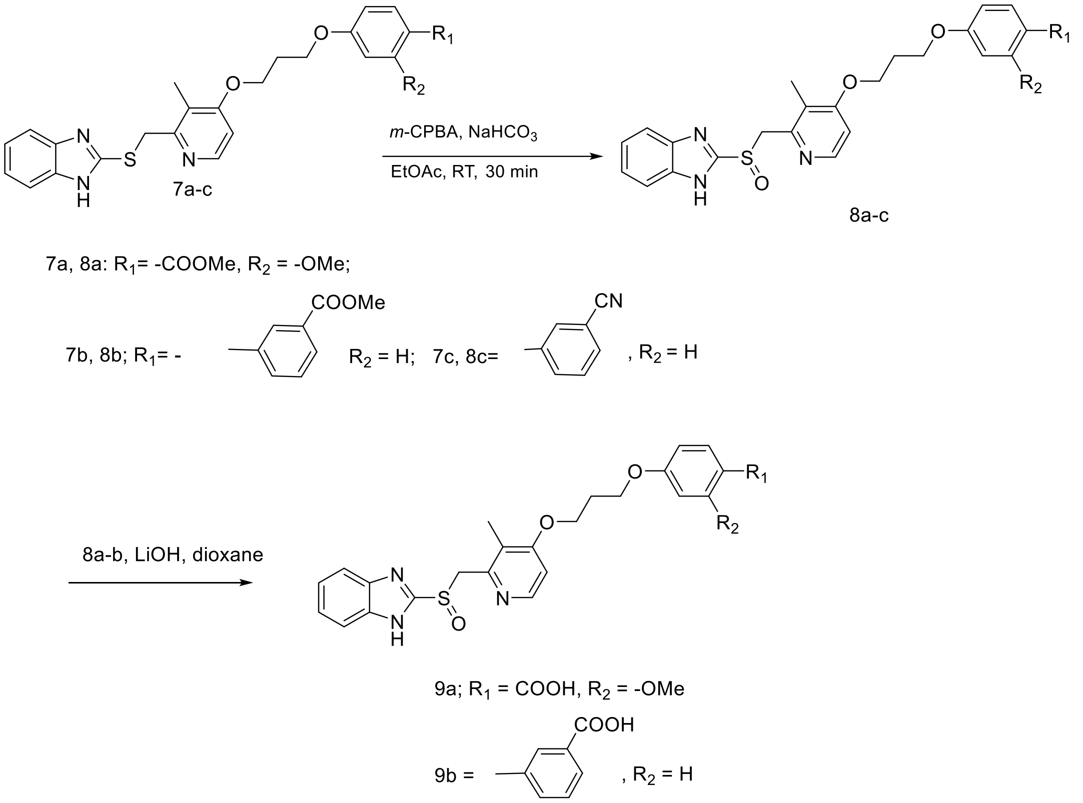

Scheme II

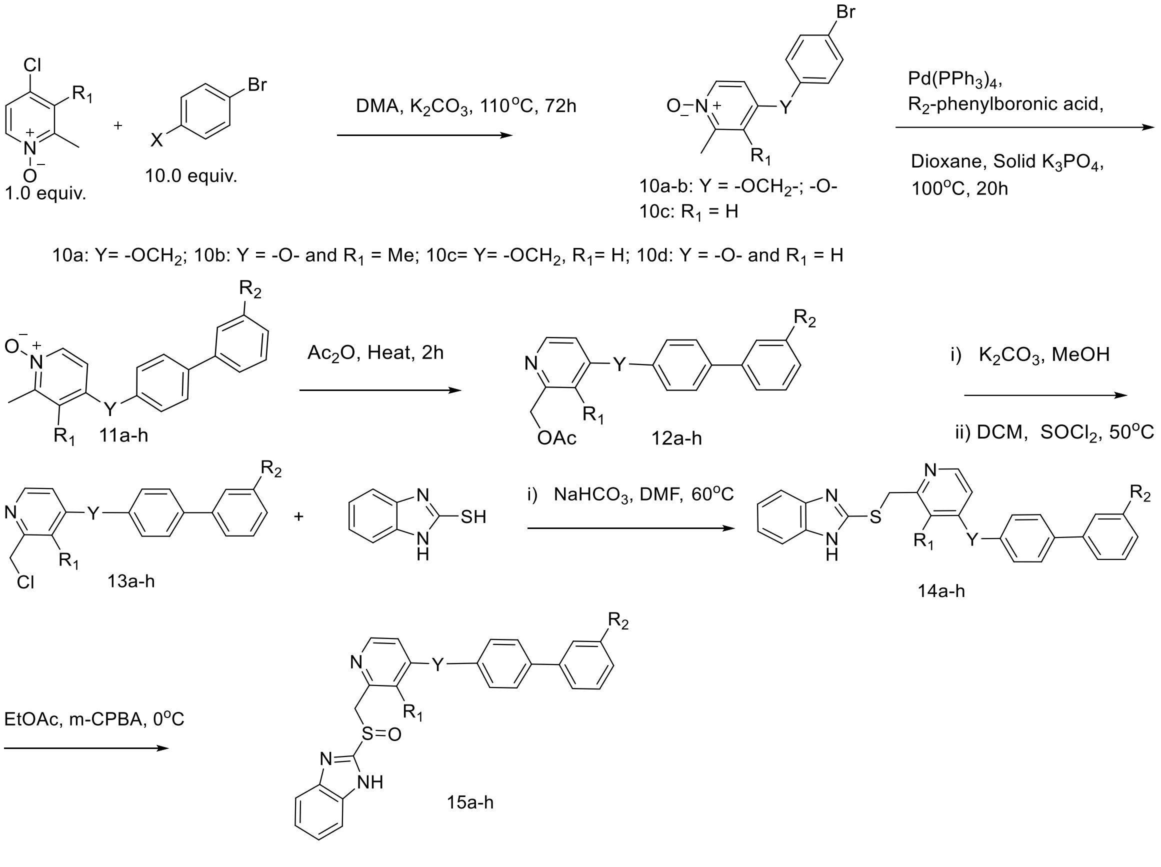

11a to 15a: Y= -OCH_2, R_1-Me, R_2 = -COOMe, 11b to 15b: Y= -OCH_2, R_1-Me, R_2 = -CN, 11c, to 15c: Y= -O-, R_1-Me, R_2 = -COOMe, 11d to 15d: Y= -O-, R_1-Me, R_2 = -CN, 11e to 15e: Y= -OCH_2, R_1-H, R_2 = -COOMe, 11f to 15f: Y= -OCH_2, R_1-H, R_2 = -CN, 11g to 15g: Y= -O-, R_1-H, R_2 = -COOMe, 11h to 15h: Y= -O-, R_1-H, R_2 = -CN

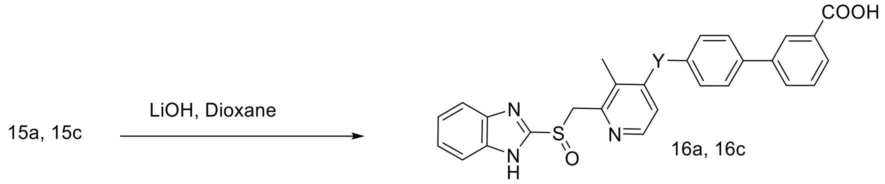

16a: Y = -OCH_2-, 16c: Y = -O-

Scheme III

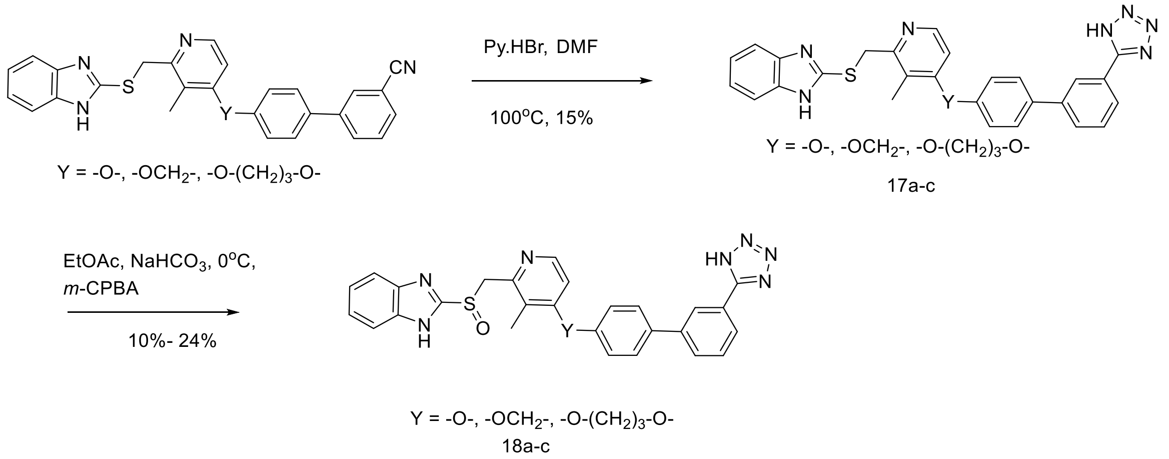

General Procedure for Preparation of 3

To a solution of the N-oxide 1[35] (1.0 mmol), and the phenol/alcohol (1.5 mmol) in anhydrous THF (5.0 ml) was added triphenylphosphine (1.6 mmol) and stirred at 0°C under argon. Diisoproyl azodicarboxylate was added dropwise over 5 min to the stirred solution at 0ºC, and allowed to come to RT, and stirring was continued for 20h more. The reaction was quenched with 10.0 ml of water and extracted with 3x25.0 ml of EtOAc. The combined organic layers were washed with water (3 x25 ml), and dried (sodium sulfate). The solution was filtered, concentrated under reduced pressure, and the residue was purified by flash silica column (40.0 g). Elution with 2% methanol in DCM, yielded the products as colorless gum. Yields: 25-45%

Alkylation of 4-Chloro-2,3-dimethylpyridine-N-oxide

A solution of the alcohol/phenol (5.0 mmol) was dissolved in 5.0 ml of anhydrous N,N-Dimethylacetamide (5.0 ml), and sodium hydride (60% in oil) was added (5.0 mmol), and stirred under argon for 30 min. Solid 4-chloro-2,3-dimethylpyridine-1-oxide (1.0 mmol) was added and heated to 110ºC for 72h. the reaction mixture was diluted with ethyl acetate (25.0 ml) and filtered through a pad of celite. The filtrate was concentrated under reduced pressure to a paste, and the crude product was purified by a flash silica column (40.0g). Elution with 12% methanol in DCM yielded the products as colorless gum. Yields: 5-10%

General procedure for Suzuki coupling of 10a-c with the aryl boronic acids[36]

To N-oxides 10a-c (1.0 mmol), and aryl boronic acid (3.0 mmol) in anhydrous dioxane (10.0 ml), was added anhydrous solid potassium phosphate (18.0 mmol) and stirred while being degassed by bubbling argon through the solution. Tetrakistriphenylphosphine palladium (5 mol%) was then added and heated to 100ºC with stirring under argon for 20h. The reaction mixture was diluted with 50.0 ml of ethyl acetate, filtered, and the filtrate was concentrated to a paste that was purified by flash silica gel (120.0g). Elution with 5% methanol in dichloromethane yielded the products as reddish-brown gum.

Boekelheide Rearrangement of N-oxides 3 & 11

The N-oxide derivatives were dissolved in (1.0 mmol) 5.0 ml of acetic anhydride and heated to 110ºC with stirring for 4h. All the volatiles were removed under reduced pressure and the crude acetates were taken to next step without further purification.
Hydrolysis of acetates 4 & 12:
The acetates were dissolved in methanol (5.0 ml for 1.0 mmol), and solid potassium carbonate (1.38g, 10.0 mmol) was added, and stirred at RT until LC/MS indicated the complete consumption of the acetates.   The solution was filtered, concentrated under reduced pressure, and the residue was purified by flash column chromatography. Elution with 5% methanol in DCM yielded the 2-hydroxymethyl derivatives as colorless gum. In most cases, the crude alcohol was taken to next step without any further purification.

Conversion of 2-hydroxymethylpyridyl derivatives to the corresponding chloro-derivatives

The hydroxymethyl derivatives from 4 & 12 (1.0 mmol, crude) were dissolved in 5.0 ml of anhydrous dichloromethane followed by the addition of 0.5 ml of thionyl chloride. The reaction mixture was heated to reflux under argon for 4h. All the volatiles were removed, and the residues were co-evaporated with toluene (2 x 5 ml) at RT and under reduced pressure to remove traces of thionyl chloride, triturated with anhydrous ethyl acetate (3x5.0 ml), decanted, and the remaining solid residue was taken to nest step without further purification.

Alkylation of chloromethyl pyridine derivatives 5 & 13 with 2-thiobenzimidazole (7a-c & 14a-h)

The crude chloro-hydrochloride salt (1.0 mmol) was heated with 2-thiobenzimidazole (1.5 mmol) in anhydrous DMF (10.0 ml) at 60ºC, and in the presence of solid sodium bicarbonate (5.0 mmol) under argon, and with stirring. After 20h, the mixture was diluted with 50.0 ml of ethyl acetate, filtered and the filtrate was concentrated under reduced pressure to a paste. The crude paste was purified by silica column (40.0g). Elution with 20% ethyl acetate in chloroform yielded the products as pale-yellow foam. Yield: 45-50%.

Oxidation of sulfides to sulfoxides
The sulfides 7 & 14 (0.1 mmol) were dissolved in ethyl acetate (5.0 ml), and solid sodium bicarbonate (0.138 g, 1.0 mol) was added and stirred at 0ºC. *m*-CPBA (77%, 26.0 mg, 0.15 mmol) was added in portions (5.0 mg at a time) over 5.0 min. After 30 min at RT, the reaction mixture was filtered, washed with ethyl acetate (3x 5.0 ml) and the combined filtrates were concentrated and purified by preparative HPLC.

Conversion of cyano substituted derivative to the corresponding tetrazole 17
The cyano-derivative (0.2 mmol) and pyridine hydrochloride (2.0 mmol), and sodium azide (4.0 mmol) were heated with stirring at 70ºC in anhydrous DMF (5.0 ml) for 20h. The crude reaction mixture was diluted with 25.0 ml of water and extracted with 3 x 25.0 ml of ethyl acetate. The combined organic layers were washed with water (2 x 20.0 ml) and dried (sodium sulfate). The solution was filtered, concentrated and the residue was purified by flash silica column (24.0 g). Elution with dichloromethane/methanol with 0.1% ammonium hydroxide (v/v; 85:15) yielded the products as off- white solids. Yield: 20-30%
Conversion of tetrazole-sulfides 17a-c to the sulfoxides 18a-c:
Repeated as described above for other sulfoxides and purified by silica gel column. Yields: 20-30%

Hydrolysis of sulfoxide methyl esters to acids (9a, 16a & 16c)
The sulfoxide-ester (0.1 mmol) in dioxane (200uL) was cooled in an ice bath, and 1M LiOH in methanol (1.0 ml, 1.0 mmol) was added and stirred until all the starting material was completely consumed (LC/MS). The reaction mixture was adjusted to pH 8.0 with acetic acid, 1.0 ml of methanol was added and filtered through 0.45µ filter. The filtrate was purified by preparative HPLC.

Synthesis of Compound 20

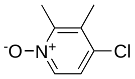

To a solution of 2,3-dimethyl-4-nitropyridine 1-oxide (1.68 g, 10 mmol) in anhydrous ethanol (4.1 ml, 70 mmol) at 0 ºC was added slowly dropwise acetyl chloride (1.8 ml, 25 mmol) over a period of 30 min. After the addition was complete ice bath was removed and reaction was heated to reflux for 5 hours. After this all the volatiles were removed in vacuo and residue was dissolved in water and basified with saturated potassium carbonate solution in water to a pH of ~ 10. Aqueous layer was then extracted with dichloromethane (3 x 15 ml), combined organic layer was dried over anhydrous sodium sulfate and concentrated. Solid residue was then crystallized from toluene/heptane to give pure desired product. Yield 1.5 g 98 %

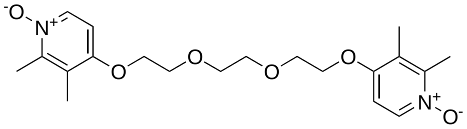

To a solution of 4-chloro-2,3-dimethylpyridine 1-oxide (314 mg, 2 mmol) and triethylene glycol (165 mg, 1.1 mmol) in 6 ml of anhydrous 1,4-dioxane (6 ml) was added powdered potassium hydroxide (224 mg, 4 mmol) and resulting reaction mixture was heated to 110 ºC for 24 h. After this time LCMS of the reaction indicated formation of product, all volatiles were removed in vacuo and residue was taken in minimum amount of water extracted with dichloromethane (5 x). Combined organic layer was dried anhydrous sodium sulfate concentrated and residue was purified over silica gel using 0 to 25 % methanol in dichloromethane to give desired product. Yield 380 mg 88 %

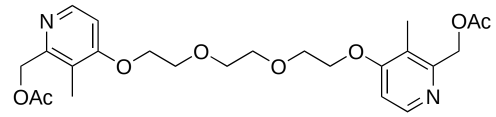

To solid 4,4'-(((ethane-1,2-diylbis(oxy))bis(ethane-2,1-diyl))bis(oxy))bis(2,3-dimethylpyridine 1-oxide) (380 mg, 0.97 mmol) was added 6 ml of acetic anhydride and resulting solution was heated to 100 oC for 12 h. Reaction was then cooled to room temperature and excess acetic anhydride was quenched by dropwise addition of methanol. All then volatiles were then removed in vacuo and residue was taken basified with saturated sodium bicarbonate solution in water and aqueous layer was extracted with dichloromethane (3 x 20 ml). Combined organic layer was dried over anhydrous sodium sulfate, concentrated and chromatographed over silica gel using 0 to 20 % methanol in dichloromethane to give desired product. Yield 328 mg, 71 %

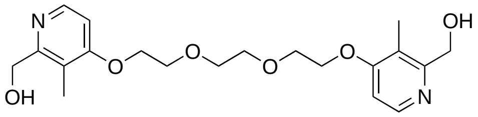

A solution of ((((ethane-1,2-diylbis(oxy))bis(ethane-2,1-diyl))bis(oxy))bis(3-methylpyridine-4,2-diyl))bis(methylene) diacetate (328 mg, 0.69 mmol) in 5 ml 6 M hydrochloric acid in water was heated to 70 ºC for 5 h. After this time LCMS indicated complete hydrolysis of starting material, all the hydrochloric acid was then removed in vacuo and residue was further dried in vacuum to give the product as HCl salt which was used in next step without purification. Yield 312 mg, 98 %

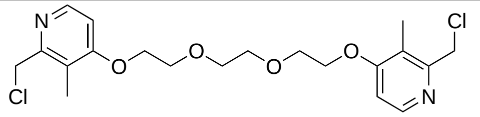

To a solution of ((((ethane-1,2-diylbis(oxy))bis(ethane-2,1-diyl))bis(oxy))bis(3-methylpyridine-4,2-diyl))dimethanol.bis hydrochloride salt (312 mg, 0.67 mmol) in 3 ml of anhydrous dichloromethane at 0 ºC was added slowly dropwise thionyl chloride (0.49 ml, 6.7 mmol). After the addition was complete ice bath was removed and reaction was allowed proceed at room temperature overnight. After this time all the volatiles were removed in vacuo and residue was basified with saturated sodium bicarbonate solution and aqueous layer was extracted with dichloromethane (3 x 10 ml). Combined organic layer was dried over anhydrous sodium sulfate, concentrated and residue was chromatographed over silica gel using 0 to 10 % methanol in dichloromethane to give the product. Yield 170 mg, 60 %

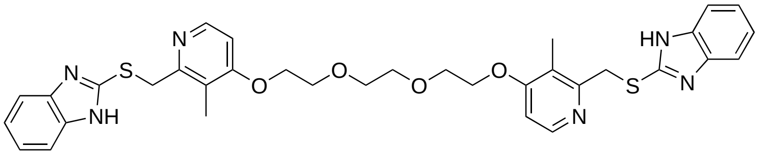

To a solution of 1,2-bis(2-((2-(chloromethyl)-3-methylpyridin-4-yl)oxy)ethoxy)ethane (170 mg, 0.4 mmol) and 2-Benzimidazolethiol (143 mg, 0.95 mmol) in 4 ml of anhydrous acetonitrile was added triethylamine (0.55 ml, 4 mmol). Resulting reaction mixture was heated to reflux overnight. All the volatiles were removed in vacuo and residue was purified by column chromatography on silica gel using 0 to 30 % methanol in dichloromethane to give desired product.

^{1}H NMR (400 MHz, cdcl$_3$) δ 12.89 (s, 2H), 8.31 (d, *J* = 5.8 Hz, 2H), 7.67 – 7.56 (m, 2H), 7.50 – 7.39 (m, 2H), 7.18 (dt, *J* = 6.1, 3.6 Hz, 4H), 6.72 (d, *J* = 5.8 Hz, 2H), 4.37 (s, 4H), 4.16 (t, *J* = 4.7 Hz, 4H), 3.88 (t, *J* = 4.7 Hz, 4H), 3.74 (s, 4H), 2.24 (s, 6H).

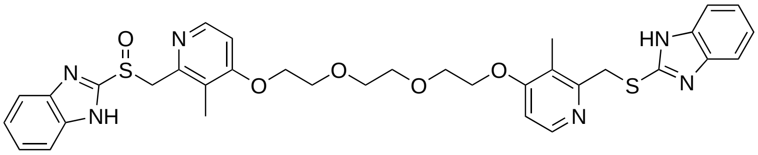

A solution of 1,2-bis(2-((2-(((1*H*-benzo[*d*]imidazol-2-yl)thio)methyl)-3-methylpyridin-4-yl)oxy)ethoxy)ethane (68 mg, 0.104 mmol) in 1.5 ml of dichloromethane and 1.5 ml of 1.5 ml water at 0 ºC was buffered with sodium bicarbonate (35 mg, 0.42 mmol). To this solution was then added m-chloroperbenzoic acid (0.052 mmol, 9 mg, 77 % purity) and resulting reaction mixture was then stirred at 0 ºC for 30 min. Reaction was then quenched with by addition of 10 % sodium sulfite solution and extracted with DCM (5 x). Organic layer was washed with saturated sodium bicarbonate solution and dried over anhydrous sodium sulfate, concentrated and chromatographed over silica gel using 0 to 20 % methanol in dichloromethane buffered with 2 % triethylamine to give the product.

^{1}H NMR (400 MHz, dmso) δ 8.21 (dd, *J* = 11.1, 5.6 Hz, 2H), 7.64 (dd, *J* = 6.0, 3.4 Hz, 2H), 7.44 (s, 2H), 7.32 – 7.24 (m, 2H), 7.16 – 7.07 (m, 2H), 6.95 (dd, *J* = 5.7, 3.0 Hz, 2H), 4.78 (d, *J* = 13.6 Hz, 1H), 4.69 (d, *J* = 13.6 Hz, 1H), 4.68 (s, 2 H), 4.24 – 4.13 (m, 4H), 3.79 (dt, *J* = 6.7, 2.5 Hz, 4H), 3.63 (s, 4H), 2.20 (s, 3H), 2.13 (s, 3H).

Prazole Derivatives

3a:

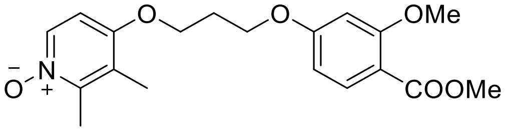

^{1}H NMR (400 MHz, cdcl$_3$) δ 8.12 (d, *J* = 7.2 Hz, 1H), 7.66 – 7.62 (m, 1H), 7.54 (d, *J* = 1.9 Hz, 1H), 6.89 (d, *J* = 8.4 Hz, 1H), 6.66 (d, *J* = 7.2 Hz, 1H), 4.32 – 4.15 (m, 4H), 3.88 (s, 6H), 2.37 (p, *J* = 6.0 Hz, 2H), 2.17 (s, 3H).
^{13}C NMR (101 MHz, cdcl$_3$) δ 166.75, 154.75, 152.03, 148.91, 137.15, 132.03, 128.41, 123.42, 123.12, 112.38, 111.66, 105.59, 64.91, 55.97, 52.01, 28.85, 14.31, 11.95.
M.S.: [M+H] 362.2

3b:

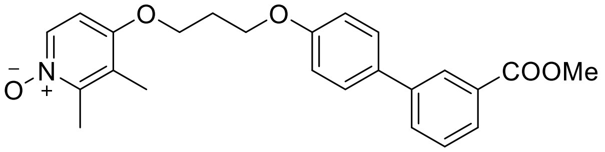

^{1}H NMR (400 MHz, cdcl$_3$) δ 8.22 (d, *J* = 1.8 Hz, 1H), 8.14 (d, *J* = 7.2 Hz, 1H), 7.96 (dt, *J* = 7.6, 1.1 Hz, 1H), 7.73 (ddd, *J* = 7.8, 2.0, 1.0 Hz, 1H), 7.55 (dt, *J* = 8.6, 2.0 Hz, 2H), 7.48 (t, *J* = 7.7 Hz, 1H), 6.99 (dt, *J* = 8.6, 2.1 Hz, 2H), 6.67 (d, *J* = 7.2 Hz, 1H), 4.21 (td, *J* = 6.0, 3.8 Hz, 4H), 3.93 (d, *J* = 1.0 Hz, 3H), 2.53 (s, 3H), 2.34 (p, *J* = 6.0 Hz, 2H), 2.19 (s, 3H).
^{13}C NMR (101 MHz, cdcl$_3$) δ 167.11, 158.51, 155.06, 149.04, 140.89, 137.02, 132.95, 131.04, 130.65, 128.82, 128.27, 127.81, 123.21, 114.81, 105.59, 65.28, 64.06, 52.18, 29.02, 14.33, 11.98.
M.S.: [M+H] 408.2

3c:

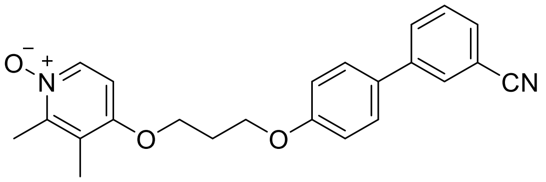

^{1}H NMR (400 MHz, DMSO) δ 8.10 – 8.03 (m, 1H), 7.98 – 7.92 (m, 1H), 7.73 (dt, J = 7.7, 1.3 Hz, 1H), 7.70 – 7.65 (m, 1H), 7.60 (t, J = 7.8 Hz, 1H), 7.08 – 7.02 (m, 1H), 4.19 (t, J = 6.1 Hz, 4H), 2.32 (s, 3H), 2.19 (m, J = 5.8 Hz, 2H), 2.11 (s, 3H).
^{13}C NMR (101 MHz, DMSO) δ 159.30, 153.72, 148.03, 141.37, 136.98, 131.39, 130.86, 130.57, 130.09, 128.65, 123.05, 119.41, 115.57, 115.48, 112.54, 107.25, 65.76, 64.83, 28.90, 14.40, 12.16.
M.S. [M+H] 375.2

7a (Compound 19):

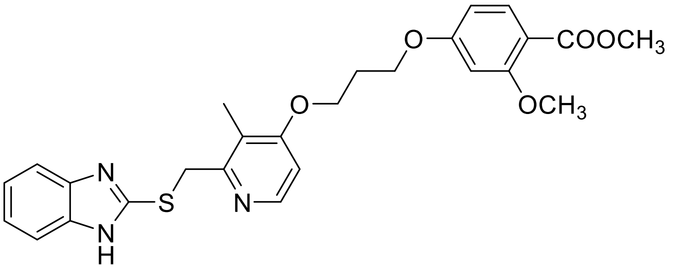

^{1}H NMR (400 MHz, cdcl$_3$) δ 8.35 (d, *J* = 5.7 Hz, 1H), 7.65 (dd, *J* = 8.4, 2.0 Hz, 1H), 7.57 – 7.49 (m, 3H), 7.22 – 7.13 (m, 2H), 6.90 (d, *J* = 8.5 Hz, 1H), 6.80 (d, *J* = 5.8 Hz, 1H), 4.37 (s, 2H), 4.28 (td, *J* = 6.0, 2.6 Hz, 4H), 3.89 (2s, 6H), 2.40 (p, *J* = 6.0 Hz, 2H), 2.25 (s, 3H).
^{13}C NMR (101 MHz, cdcl$_3$) δ 166.79, 164.09, 156.75, 152.07, 151.64, 148.94, 147.27, 123.45, 123.09, 121.77, 121.04, 112.42, 111.74, 106.15, 77.32, 77.20, 77.00, 76.68, 65.16, 64.94, 55.97, 52.01, 34.78, 28.84, 10.74.
M. S.: [M=H] 510.2
M.S. [M=H] 510.2

7b:

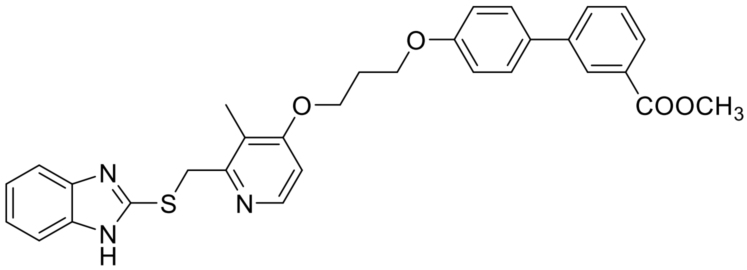

^{1}H NMR (400 MHz, cdcl$_3$) δ 8.35 (d, *J* = 5.7 Hz, 1H), 7.64 (ddd, *J* = 12.4, 8.5, 3.4 Hz, 2H), 7.54 (d, *J* = 2.0 Hz, 1H), 7.48 – 7.41 (m, 1H), 7.20 (s, 0H), 6.91 (d, *J* = 8.4 Hz, 1H), 6.81 (d, *J* = 5.8 Hz, 1H), 4.37 (s, 2H), 4.28 (td, *J* = 6.1, 2.2 Hz, 4H), 3.89 (s, 6H), 2.25 (s, 3H).
^{13}C NMR ^{13}C NMR (101 MHz, CDCl$_3$) δ 167.15, 163.53, 158.59, 153.52, 149.36, 148.35, 140.96, 132.92, 131.09, 130.67, 128.84, 128.29, 127.81, 122.98, 114.87, 106.21, 64.85, 64.16, 60.73, 52.21, 29.01, 11.18.
M.S. [M+H] 540.2

7c:

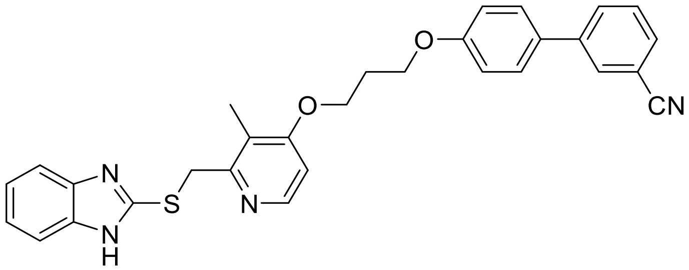

1H NMR (400 MHz, CDCl$_3$) δ 8.39 (d, *J* = 5.7 Hz, 1H), 7.83 (t, *J* = 1.8 Hz, 1H), 7.77 (dt, *J* = 7.9, 1.6 Hz, 1H), 7.67 – 7.52 (m, 5H), 7.25 – 7.16 (m, 2H), 7.06 – 6.98 (m, 2H), 6.83 (d, *J* = 5.8 Hz, 1H), 4.40 (s, 2H), 4.33 – 4.21 (m, 4H), 2.39 (p, *J* = 6.0 Hz, 2H), 2.30 (s, 3H).
13C NMR (101 MHz, CDCl$_3$) δ 164.15, 159.02, 156.78, 151.66, 147.31, 141.89, 131.64, 130.99, 130.15, 129.58, 128.27, 121.81, 121.09, 118.94, 115.05, 112.93, 106.15, 64.99, 64.16,50.87, 34.81, 29.00, 10.81.
M.S. 507.2 [M+H]

8a (Compound 14):

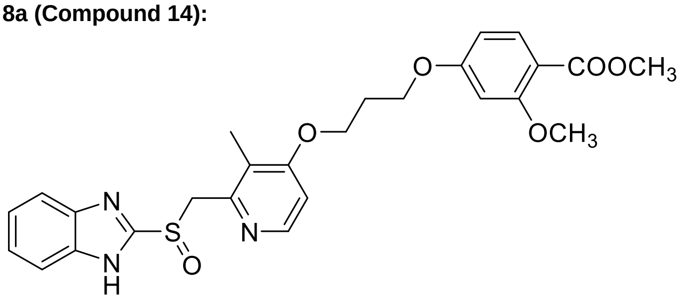

^{1}H NMR (400 MHz, DMSO) δ 7.58 (dd, *J* = 8.4, 2.0 Hz, 1H), 7.45 (d, *J* = 2.0 Hz, 1H), 7.30 (s, 2H), 7.12 (d, *J* = 8.5 Hz, 1H), 7.01 (d, *J* = 5.7 Hz, 1H), 4.86 – 4.64 (m, 2H), 4.23 (t, *J* = 6.1 Hz, 4H), 3.81 (2s, 6H), 2.25 (p, *J* = 6.0 Hz, 2H), 2.14 (s, 3H).
^{13}C NMR (101 MHz, DMSO) δ 166.42, 163.20, 154.77, 152.56, 150.60, 149.03, 148.59, 123.58, 122.41, 112.63, 112.30, 106.94, 65.55, 65.28, 60.63, 56.03, 52.38, 28.76, 11.15.
M.S.: 510.3 [M+H]; t_R:3.22 min (Analytical HPLC conditions B)

8b (Compound 16):

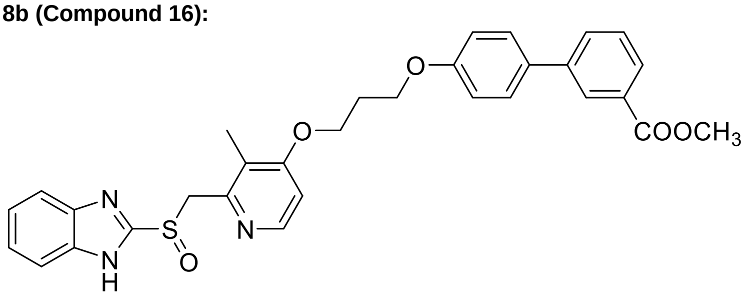

^{1}H NMR (400 MHz, CDCl$_3$) δ 8.24 (d, *J* = 5.6 Hz, 1H), 8.15 (t, *J* = 1.8 Hz, 1H), 7.89 (dt, *J* = 7.7, 1.4 Hz, 1H), 7.66 (ddd, *J* = 7.8, 2.0, 1.2 Hz, 1H), 7.52 – 7.44 (m, 2H), 7.27 – 7.19 (m, 2H), 6.68 (d, *J* = 5.7 Hz, 1H), 4.60 (d, *J* = 13.7 Hz, 1H), 3.86 (s, 3H), 2.24 (p, *J* = 6.0 Hz, 2H).
^{13}C NMR (101 MHz, CDCl$_3$) δ 167.15, 163.53, 158.59, 153.52, 149.36, 148.35, 140.96, 132.92, 131.09, 130.67, 128.84, 128.29, 127.81, 122.98, 114.87, 106.21, 64.85, 64.16, 60.73, 52.21, 29.01, 11.18.
t_R: 3.582 min; 50-100 over 5.0 min; **M.S.** 556.2 [M+H] (Analytical HPLC conditions B)

8c (Compound 15):

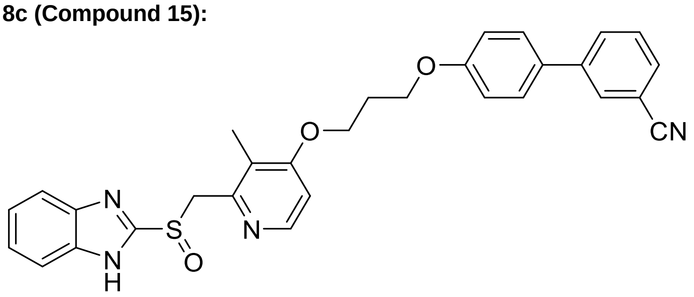

^{1}H NMR (400 MHz, cdcl$_3$) δ 8.24 (d, *J* = 5.6 Hz, 1H), 7.77 – 7.66 (m, 2H), 7.51 (d, *J* = 7.6 Hz, 1H), 7.25 (dt, *J* = 6.2, 3.6 Hz, 2H), 6.93 (d, *J* = 8.6 Hz, 2H), 6.69 (d, *J* = 5.7 Hz, 1H), 4.74 (d, *J* = 13.7 Hz, 1H), 4.61 (d, *J* = 13.7 Hz, 1H), 4.14 (dt, *J* = 11.3, 5.9 Hz, 4H), 2.25 (p, *J* = 6.0 Hz, 2H), 2.11 (s, 3H).
^{13}C NMR (101 MHz, cdcl$_3$) δ 163.48, 159.03, 153.47, 149.35, 148.34, 141.90, 131.58, 130.98, 130.21, 130.10, 129.55, 128.24, 122.95, 118.92, 115.05, 112.89, 106.18, 64.77, 64.18, 60.73, 28.96, 11.16.
t$_R$: 6.19 min; **M.S.** 523.2 [M+H] (Analytical HPLC conditions A)

9a:

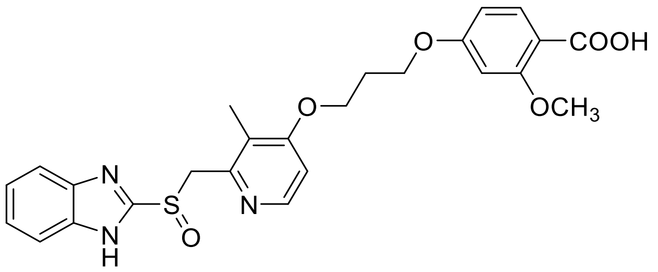

^{1}H NMR (400 MHz, D$_2$O) δ 7.87 (d, *J* = 5.6 Hz, 1H), 7.40 – 7.25 (m, 4H), 7.05 (dd, *J* = 6.2, 3.1 Hz, 2H), 6.68 (d, *J* = 8.6 Hz, 1H), 6.57 (d, *J* = 5.8 Hz, 1H), 4.59 (d, *J*= 13.0Hz), 1H), 4.48 (d, *J* = 13.0 Hz, 1H), 3.93 – 3.84 (m, 4H), 3.61 (s, 3H), 1.96 (s, 2H), 1.45 (s, 3H).
^{13}C NMR (101 MHz, D$_2$O) δ 173.90, 163.98, 159.87, 149.89, 147.98, 147.71, 147.43, 129.82, 124.34, 123.61, 123.03, 116.21, 112.59, 112.10, 107.03, 65.53, 65.19, 55.55, 27.93, 10.26.
t$_R$: 3.3 min; **M.S.** 496.3 [M+H]; (Analytical HPLC Conditions A).

9b (Compound 17):

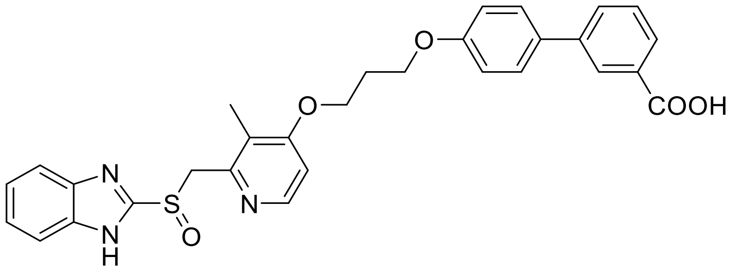

^{1}H NMR (400 MHz, DMSO) δ 8.16 (d, *J* = 5.6 Hz, 1H), 8.06 (t, *J* = 1.8 Hz, 1H), 7.78 (dt, *J* = 7.7, 1.3 Hz, 1H), 7.66 (dt, *J* = 8.0, 1.4 Hz, 1H), 7.63 – 7.50 (m, 4H), 7.40 (t, *J* = 7.7 Hz, 1H), 7.21 (dd, *J* = 6.1, 3.2 Hz, 2H), 7.02 – 6.91 (m, 3H), 4.75 (d, *J* = 13.6 Hz, 1H), 4.63 (d, *J* = 13.6 Hz, 1H), 4.16 (dt, *J* = 15.8, 6.1 Hz, 4H), 2.17 (p, *J* = 6.2 Hz, 2H), 2.09 (s, 3H).
^{13}C NMR (101 MHz, DMSO) δ 168.31, 163.19, 158.64, 155.03, 150.73, 148.57, 139.98, 132.72, 129.36, 129.08, 128.28, 127.25, 123.45, 122.38, 115.44, 106.92, 65.32, 64.71, 60.47, 28.82, 11.17.
t$_R$: 3.19 min; **M.S.** 542.2 [M+Na] (Analytical HPLC Conditions B)

11c:

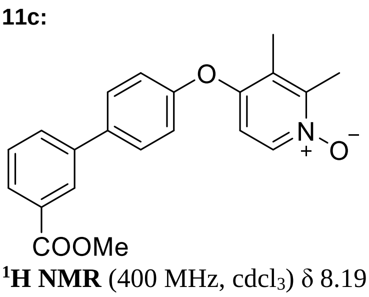

^{1}H NMR (400 MHz, cdcl$_3$) δ 8.19 (t, *J* = 1.8 Hz, 1H), 8.12 (d, *J* = 7.2 Hz, 1H), 7.96 (dt, *J* = 7.8, 1.4 Hz, 1H), 7.76 – 7.64 (m, 1H), 7.65 – 7.56 (m, 2H), 7.46 (t, *J* = 7.7 Hz, 1H), 7.15 – 7.03 (m, 2H), 6.80 (d, *J* = 3.3 Hz, 1H), 6.72 (dd, *J* = 7.2, 3.4 Hz, 1H), 3.88 (s, 3H), 2.44 (s, 3H).
^{13}C NMR (101 MHz, cdcl$_3$) δ 166.87, 155.51, 154.20, 150.39, 140.40, 140.20, 137.51, 131.30, 130.85, 129.06, 129.02, 128.90, 128.58, 128.11, 126.97, 120.65, 120.55, 114.73, 112.68, 77.35, 77.24, 77.04, 76.72, 52.25, 18.29.

M.S. [M+H] 350.2

15a (Compound 10):

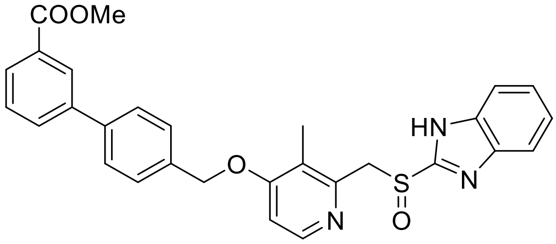

^{1}H NMR (400 MHz, DMSO) δ 8.21 – 8.11 (m, 1H), 7.95 – 7.87 (m, 1H), 7.69 (d, *J* = 8.0 Hz, 1H), 7.61 – 7.48 (m, 2H), 7.23 (dt, *J* = 7.2, 3.6 Hz, 1H), 7.02 (d, *J* = 5.7 Hz, 0H), 5.22 (s, 1H), 4.76 (d, *J* = 13.7 Hz, 0H), 4.66 (d, *J* = 13.7 Hz, 0H), 3.83 (s, 1H), 2.14 (s, 1H).
^{13}C NMR (101 MHz, DMSO) δ 166.58, 163.02, 154.83, 150.81, 148.53, 140.71, 139.22, 136.57, 132.00, 130.84, 130.03, 128.65, 127.50, 122.54, 107.47, 69.61, 60.61, 52.74, 11.34.
t_R: 4.56 min; **M.S.** 512.4 [M+H] (Analytical HPLC conditions B)

15b (Compound 11):

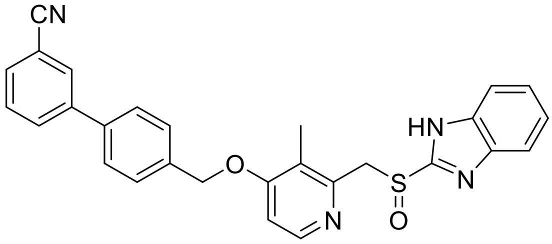

^{1}H NMR (400 MHz, DMSO) δ 8.18 (d, *J* = 5.6 Hz, 1H), 8.11 (d, *J* = 1.9 Hz, 1H), 7.98 (dt, *J* = 8.0, 1.4 Hz, 1H), 7.80 – 7.70 (m, 3H), 7.65 – 7.48 (m, 5H), 7.23 (dt, *J* = 7.1, 3.5 Hz, 2H), 7.01 (d, *J* = 5.7 Hz, 1H), 5.22 (s, 2H), 4.76 (d, *J* = 13.6 Hz, 1H), 4.66 (d, *J* = 13.7 Hz, 1H), 2.14 (s, 3H).
^{13}C NMR (101 MHz, DMSO) δ 163.00, 154.89, 150.84, 148.52, 141.28, 138.17, 136.99, 131.97, 131.62, 130.72, 130.63, 128.67, 127.63, 123.56, 122.53, 119.24, 112.58, 107.45, 69.54, 60.61, 11.34.
t_R: 4.39 min; **M.S.** 477.2 [M-H]

15c (Compound 5):

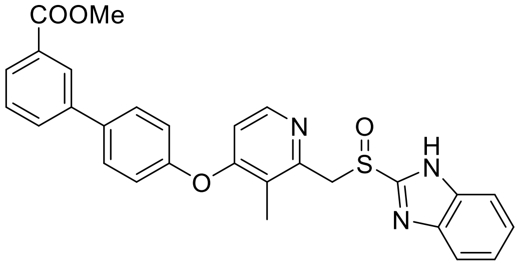

^{1}H NMR (400 MHz, DMSO) δ 8.23 – 8.16 (m, 1H), 8.13 (s, 1H), 7.90 (s, 3H), 7.80 (d, *J* = 7.9 Hz, 0H), 7.72 (d, *J* = 8.2 Hz, 2H), 7.59 (d, *J* = 8.2 Hz, 4H), 7.46 (d, *J* = 8.6 Hz, 0H), 7.28 – 7.22 (m, 2H), 7.12 (d, *J* = 8.1 Hz, 2H), 6.81 (d, *J* = 8.4 Hz, 0H), 6.65 (d, *J* = 5.7 Hz, 1H), 4.89 – 4.80 (m, 1H), 4.80 – 4.71 (m, 1H), 3.83 (s, 3H), 2.20 (s, 3H).
^{13}C NMR (101 MHz, DMSO) δ 166.61, 162.53, 155.04, 154.68, 152.50, 148.68, 140.24, 136.14, 131.89, 130.87, 130.08, 129.30, 128.54, 127.53, 124.34, 123.68, 120.74, 111.37, 60.54, 52.77, 11.50.
t_R: 4.5 min; **M.S.** 498.4 [M+H] (Analytical HPLC conditions B)

15d (Compound 4):

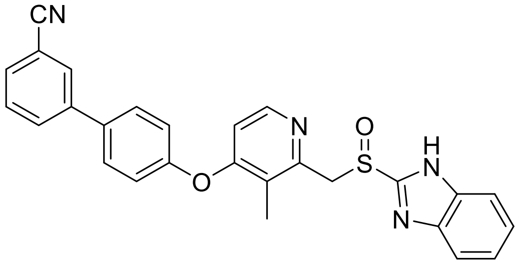

^{1}H NMR (400 MHz, DMSO) δ 8.24 (dd, *J* = 5.6, 1.4 Hz, 1H), 8.16 (q, *J* = 1.7 Hz, 1H), 8.03 (dq, *J* = 8.1, 1.6 Hz, 1H), 7.82 (ddd, *J* = 7.4, 3.6, 1.5 Hz, 3H), 7.71 – 7.61 (m, 3H), 7.28 (ddd, *J* = 6.3, 3.3, 1.4 Hz, 2H), 7.22 – 7.14 (m, 2H), 6.70 (dd, *J* = 5.6, 1.4 Hz, 1H), 4.90 (dd, *J* = 13.6, 1.4 Hz, 1H), 4.79 (dd, *J* = 13.6, 1.4 Hz, 1H), 2.26 (d, *J* = 1.4 Hz, 3H). **^{13}C NMR** (101 MHz, DMSO) δ 155.40, 154.89, 152.60, 148.66, 140.76, 135.00, 131.47, 130.65, 129.44, 124.40, 123.51, 120.64, 119.24, 112.60, 111.45, 60.51, 11.49.

t_R: 3.1 min; **M.S.** 465.4 (Analytical HPLC conditions B).

15e (Compound 13):

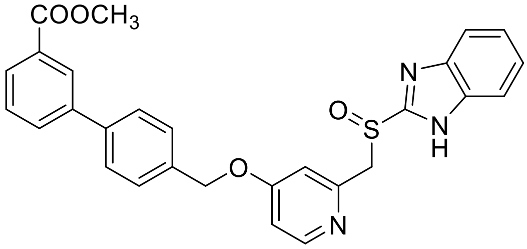

¹H NMR (400 MHz, DMSO) δ 8.28, 8.27, 8.26, 8.14, 8.13, 8.13, 7.91, 7.90, 7.89, 7.88, 7.67, 7.67, 7.65, 7.62, 7.61, 7.60, 7.59, 7.58, 7.58, 7.56, 7.54, 7.44, 7.42, 7.26, 7.25, 7.24, 7.23, 7.23, 7.22, 6.95, 6.94, 6.94, 6.93, 6.87, 6.87, 5.05, 5.02, 5.02, 4.99, 4.68, 4.65, 4.59, 4.55, 3.82.
¹³C NMR (101 MHz, DMSO) δ 166.61, 165.10, 154.38, 152.83, 151.39, 140.66, 139.35, 136.10, 132.02, 129.09, 128.71, 127.45, 123.68, 112.51, 110.31, 69.35, 62.45, 52.76.
t_R: 4.41 min (HPLC Analytical Conditions B); **M.S.**: [M+H] 498.2, [M-H] 496.2

15f:

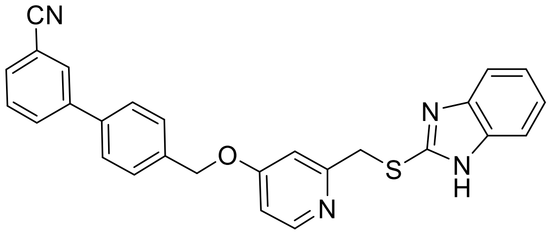

M.S.: 488.14

15g (Compound 8):

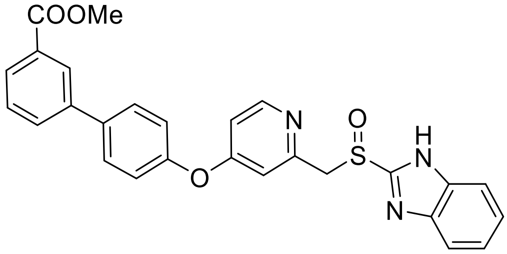

¹H NMR (400 MHz, DMSO) δ 8.36 (d, *J* = 5.8 Hz, 1H), 8.13 (s, 1H), 7.90 (t, *J* = 7.1 Hz, 2H), 7.65 – 7.54 (m, 5H), 7.28 – 7.21 (m, 2H), 7.05 (d, *J* = 8.1 Hz, 2H), 6.90 (dd, *J* = 5.7, 2.4 Hz, 1H), 6.74 (d, *J* = 2.4 Hz, 1H), 4.64 (q, *J*=12.0 Hz, 2H) 3.84 (s, 3H).
¹³C NMR (101 MHz, DMSO) δ 166.61, 164.80, 154.10, 153.69, 153.28, 151.93, 140.12, 136.71, 131.90, 130.87, 130.09, 129.27, 128.63, 127.60, 123.70, 121.44, 113.51, 112.16, 62.13, 52.79.
tR: 4.63 min (HPLC Analytical Conditions B); **M.S.**: [M+H] 484.2

15h (Compound 9):

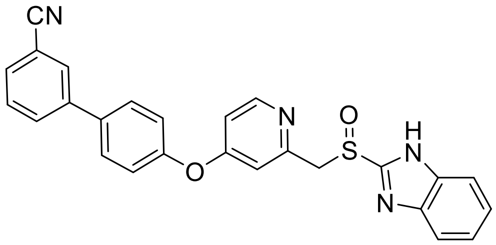

¹H NMR (400 MHz, DMSO) δ 8.36 (d, *J* = 5.7 Hz, 1H), 8.10 (t, *J* = 1.8 Hz, 1H), 7.78 (d, *J* = 7.7 Hz, 1H), 7.75 – 7.67 (m, 2H), 7.67 – 7.52 (m, 3H), 7.12 – 7.05 (m, 2H), 6.89 (dd, *J* = 5.7, 2.5 Hz, 1H), 6.77 (d, *J* = 2.4 Hz, 1H), 4.71 – 4.56 (m, 2H).
¹³C NMR (101 MHz, DMSO) δ 164.61, 154.15, 153.37, 151.96, 140.67, 135.63, 131.88, 131.60, 130.65, 129.45, 123.66, 121.54, 119.26, 113.68, 112.63, 112.13, 62.12.
t_R: 4.39 min ((HPLC Analytical Conditions B); **M.S.**: [M+H] 451.2

16a (Compound 12):

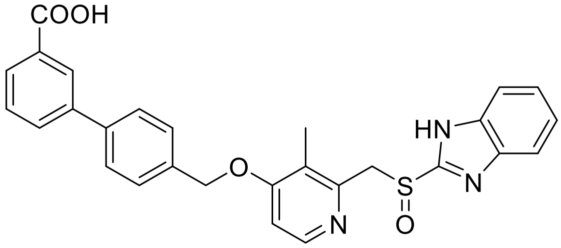

^{1}H NMR (400 MHz, DMSO) δ 8.18 (d, *J* = 5.6 Hz, 1H), 8.13 (s, 1H), 7.86 (d, *J* = 7.7 Hz, 1H), 7.79 (d, *J* = 7.7 Hz, 1H), 7.67 (d, *J* = 7.9 Hz, 2H), 7.59 (dd, *J* = 6.1, 3.3 Hz, 2H), 7.53 – 7.44 (m, 4H), 7.23 (dd, *J* = 6.2, 3.2 Hz, 2H), 7.02 (d, *J* = 5.7 Hz, 1H), 5.21 (s, 2H), 4.76 (d, *J* = 13.6 Hz, 1H), 4.66 (d, *J* = 13.6 Hz, 1H), 2.14 (s, 3H).**^{13}C NMR** (101 MHz, DMSO) δ 163.05, 155.19, 150.82, 148.52, 140.13, 136.22, 129.42, 128.79, 128.65, 127.76,
127.40, 123.58, 122.54, 107.46, 69.66, 60.57, 11.35.
t_R: 3.36 min; **M.S.** 498.4 [M+H]. (Analytical HPLC conditions B)

16c (Compound 6):

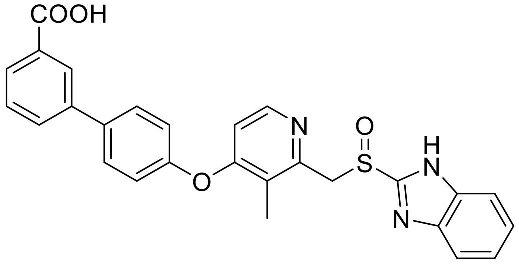

^{1}H NMR (400 MHz, DMSO) δ 8.17 (d, *J* = 5.5 Hz, 1H), 8.13 (d, *J* = 1.8 Hz, 1H), 7.87 (d, *J* = 7.7 Hz, 1H), 7.84 – 7.77 (m, 1H), 7.74 – 7.66 (m, 2H), 7.60 (dt, *J* = 6.9, 3.5 Hz, 2H), 7.50 (t, *J* = 7.7 Hz, 1H), 7.25 (dt, *J* = 6.1, 3.5 Hz, 2H), 7.14 – 7.06 (m, 2H), 6.64 (d, *J* = 5.5 Hz, 1H), 4.86 (d, *J* = 13.7 Hz, 1H), 4.76 (d, *J* = 13.6 Hz, 1H), 2.21 (s, 3H).
^{13}C NMR (101 MHz, DMSO) δ 168.18, 162.58, 154.83, 154.66, 152.48, 148.66, 139.77, 136.65, 133.95, 130.64, 129.58, 129.19, 128.69, 127.70, 124.30, 123.68, 120.71, 111.31, 60.51, 11.50.
t_R: 4.00 min; **M.S.**: 484.2 [M+H] (Analytical HPL conditions A).

17a:

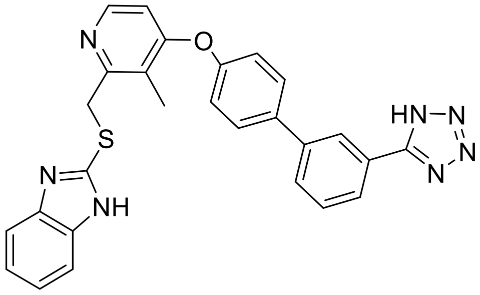

1H NMR (400 MHz, DMSO) δ 8.32 (t, J = 1.8 Hz, 1H), 8.26 (d, J = 5.7 Hz, 1H), 8.07 – 8.00 (m, 1H), 7.89 (dt, J = 7.9, 1.5 Hz, 1H), 7.85 – 7.75 (m, 2H), 7.69 (t, J = 7.8 Hz, 1H), 7.65 – 7.58 (m, 2H), 7.44 (dt, J = 7.1, 3.5 Hz, 2H), 7.16 – 7.05 (m, 3H), 5.29 (s, 2H), 2.27 (s, 3H).
^{13}C NMR (101 MHz, DMSO) δ 167.49, 155.28, 150.62, 148.10, 141.23, 139.27, 136.69, 130.61, 129.77, 128.74, 127.48, 126.46, 125.64, 121.86, 120.55, 107.39, 69.65, 36.63, 11.09.

17b:

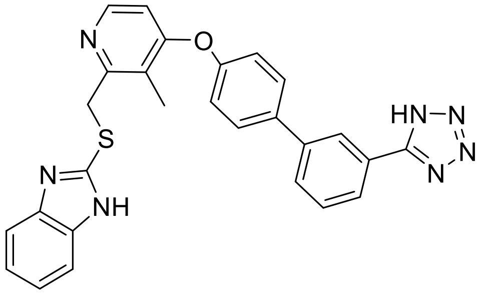

^{1}H NMR (400 MHz, DMSO) δ 8.32 (d, *J* = 5.7 Hz, 1H), 8.18 (t, *J* = 1.8 Hz, 1H), 8.05 (dt, *J* = 8.0, 1.4 Hz, 1H), 7.88 – 7.77 (m, 3H), 7.73 – 7.57 (m, 4H), 7.47 (dd, *J* = 5.9, 3.2 Hz, 2H), 7.19 – 7.10 (m, 3H), 5.32 (s, 2H), 4.75 (s, 2H), 2.30 (s, 3H).

^{13}C NMR (101 MHz, DMSO) δ 155.16, 150.49, 141.29, 138.23, 136.94, 132.00, 131.66, 130.66, 128.74, 127.66, 121.96, 120.90, 119.27, 112.60, 107.54, 69.76, 11.12.

17c:

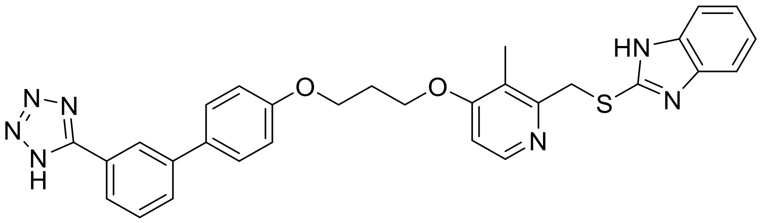

^{1}H NMR (400 MHz, DMSO) δ 8.29 (d, J = 1.8 Hz, 1H), 8.26 (d, J = 5.6 Hz, 1H), 7.78 (dt, J = 8.1, 1.4 Hz, 1H), 7.73 – 7.66 (m, 2H), 7.62 (t, J = 7.8 Hz, 1H), 7.45 (dd, J = 5.9, 3.2 Hz, 1H), 7.11 (td, J = 5.8, 2.5 Hz, 3H), 4.70 (s, 2H), 4.25 (dt, J = 10.2, 6.1 Hz, 4H), 2.25 (m, J = 6.2 Hz, 2H), 2.24 (s, 3H)
^{13}C NMR (101 MHz, DMSO) δ 163.14, 158.95, 155.18, 150.67, 148.26, 141.11, 132.13, 130.35, 128.64, 128.41, 125.54, 124.91, 121.85, 120.30, 115.53, 106.87, 65.36, 64.79, 36.70, 28.87, 10.95.

18a:

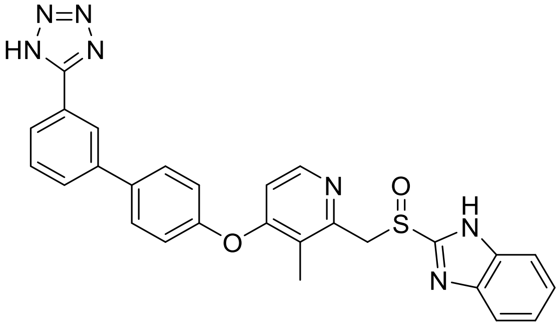

^{1}H NMR (400 MHz, DMSO) δ 8.26 (t, J = 1.9 Hz, 1H), 8.18 (d, J = 5.5 Hz, 1H), 7.97 (d, J = 7.8 Hz, 1H), 7.77 (dd, J = 7.7, 5.1 Hz, 3H), 7.60 (t, J = 7.8 Hz, 1H), 7.25 (s, 2H), 7.16 (d, J = 8.6 Hz, 2H), 6.66 (d, J = 5.6 Hz, 1H), 4.85 (d, J = 13.7 Hz, 2H), 4.76 (d, J = 13.7 Hz, 1H), 2.22 (s, 3H).
^{13}C NMR (101 MHz, DMSO) δ 162.40, 155.40, 154.89, 152.60, 148.66, 140.76, 135.00, 131.83, 131.47, 130.59, 129.44, 124.40, 123.51, 120.64, 119.24, 112.60, 111.45, 60.51, 11.49.
t_R: 4.0 min; **M.S**. 508.4 [M+H]; (Analytical HPLC conditions B)

18b (Compound 7):

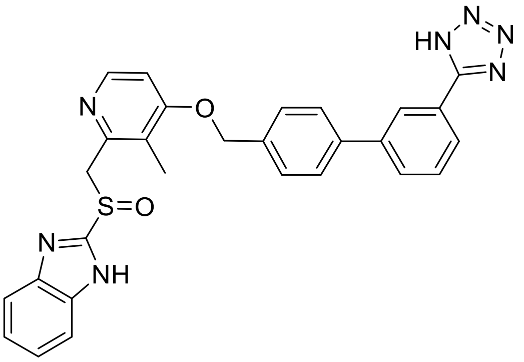

^{1}H NMR ^{1}H NMR (400 MHz, DMSO) δ 8.26 (d, J = 1.8 Hz, 1H), 8.18 (d, J = 5.6 Hz, 1H), 7.97 (dt, J = 7.7, 1.4 Hz, 1H), 7.77 – 7.68 (m, 3H), 7.63 – 7.48 (m, 5H), 7.24 (dt, J = 6.2, 3.6 Hz, 2H), 7.02 (d, J = 5.8 Hz, 1H), 5.22 (s, 2H), 4.83 – 4.62 (m, 2H), 2.14 (s, 3H).
^{13}C NMR (101 MHz, DMSO) δ 163.07, 157.49, 154.78, 150.79, 148.54, 140.91, 139.73, 136.40, 130.28, 128.71, 128.50, 128.14, 127.44, 126.18, 125.28, 122.58, 107.50, 69.69, 60.63, 11.38.
tR; 4.0; **M.S.** 522.4 [M+H] (Analytical Conditions B)

18c (Compound 18):

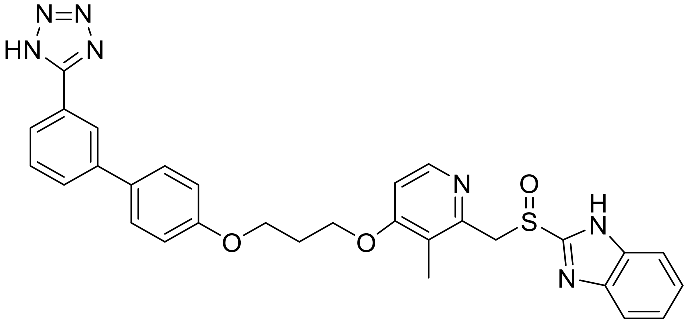

^{1}H NMR (400 MHz, DMSO) δ 8.20 – 8.13 (m, 2H), 7.88 (d, *J* = 7.6 Hz, 1H), 7.65 – 7.53 (m, 5H), 7.47 (t, *J* = 7.7 Hz, 1H), 7.23 (dd, *J* = 6.1, 3.2 Hz, 2H), 7.07 – 6.99 (m, 2H), 6.95 (d, *J* = 5.7 Hz, 1H), 4.73 (d, *J* = 13.6 Hz, 1H), 4.64 (d, *J* = 13.6 Hz, 1H), 4.17 (dt, *J* = 14.8, 6.1 Hz, 4H), 2.18 (p, *J* = 6.2 Hz, 2H), 2.09 (s, 3H).
^{13}C NMR (101 MHz, DMSO) δ 163.25, 158.92, 157.04, 154.78, 150.62, 148.61, 141.06, 140.65, 132.27, 130.26, 128.39, 127.17, 125.47, 124.84, 123.73, 122.43, 115.54, 112.46, 106.99, 65.38, 64.79, 60.64, 28.86, 11.19.
t_R: 2.834 min; **M.S.** [M+H] 566.3 (Analytical conditions A)

Cellular Screening of Efficacy for Prazole Derivatives against SARS-COV-2
Tested compounds were sent to NIAID contractors at the University of Pennsylvania for analysis according to the following protocol. Calu3 (ATCC, HTB-55) cells were pretreated with test compounds for 2 hours prior to continuous infection with SARS-CoV-2 (isolate USA WA1/2020) at a MOI=0.5. Forty-eight hours post-infection, cells were fixed, immunostained, and imaged by automated microscopy for infection (dsRNA+ cells/total cell number) and cell number. Sample well data was normalized to aggregated DMSO control wells and plotted versus drug concentration to determine the IC50 and CC50.

HIV-1 VLP Assays
293 T (ATCC CRL-3216) or HeLa (ATCC CCL-2) cell lines were used to assess susceptibility to prazole exposure as previously described[5a]. Briefly, cells were grown to 70% confluency at 37ºC in Dulbecco's modified Eagle medium supplemented with fetal bovine serum (10%) and antibiotics (1%). Unless otherwise stated in figure legends, prior to drug treatment and transfection or toxicity assays, the tissue culture media was aspirated and replaced with control or treatment media. Transfection employed XtremeGene reagent (Roche) to facilitate DNA uptake. For production of virus particles, cells were transfected with pNL4-3-ΔEnv and pIIIB Env3-1 plasmids. At 24-hr post-transfection, tissue culture media was collected and passed through a 0.45 micron filter; cells were scraped off the tissue culture plate with a rubber policeman, rinsed with PBS and pelleted. For virus isolation, the filtered media was centrifuged through a 20% sucrose cushion at 22,000 × g for 90 min at 5 °C and the pellet fraction saved for analysis. For cell lysate preparation, cell pellets were lysed with Triton X-100 buffer (50 mMTris, pH 7.4, 137 mM NaCl, 1.5 mM $MgCl_2$, 1 mM EDTA, 1% Triton X-100) containing ɨl cOmplete Protease Inhibitor (Sigma). Virus and cell lysate samples were analyzed by Western blotting. Primary antibodies used were: Rabbit anti-CA; anti-Actin (Sigma). Secondary antibodies used were: goat anti-mouse IgG Alexa Fluor 680 (Molecular Probes); goat anti-rabbit IRDye800 (Rockland). Protein bands were visualized using an infrared-based imaging system (Odyssey, LI-COR Biotechnology) and band intensities measured using the Li-Cor Odyssey software version 2.1.15.

Results

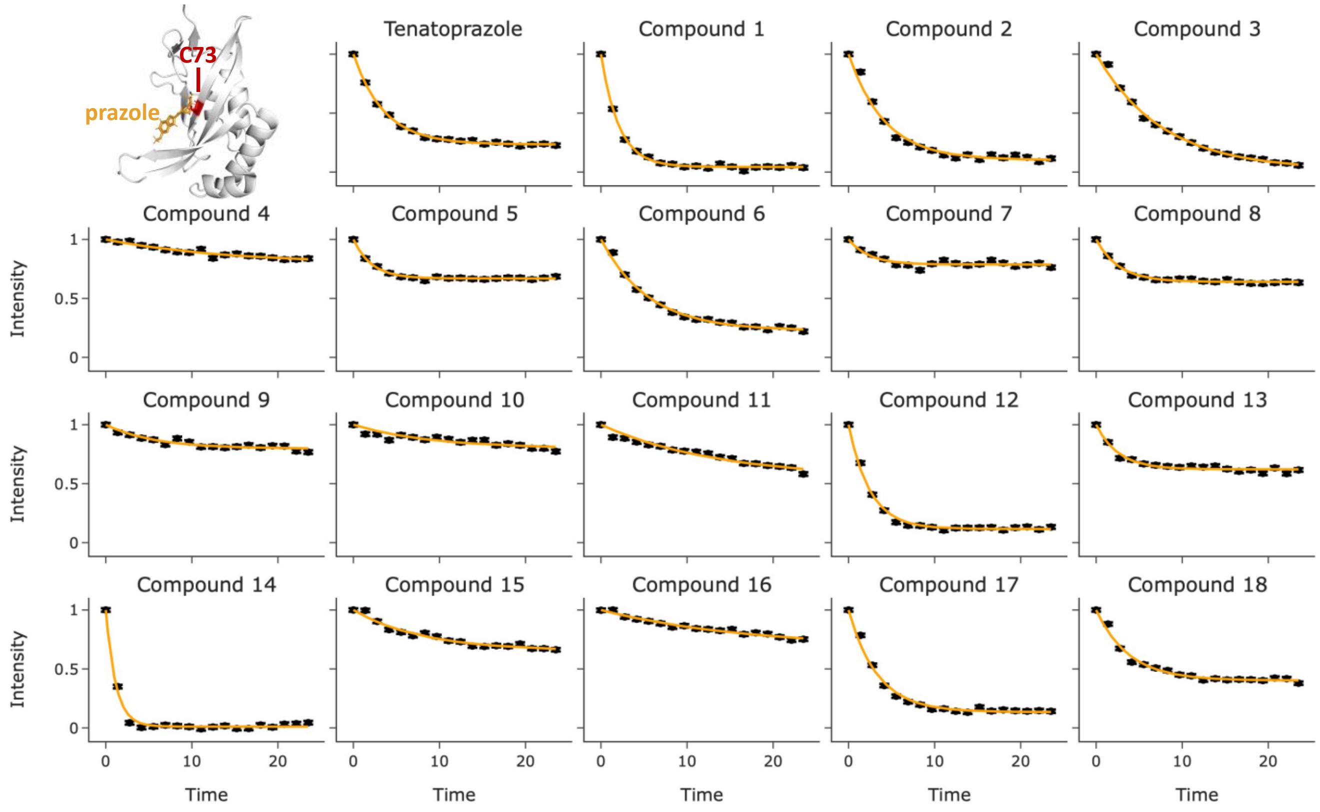

Figure S1. Monitoring prazole attachment by loss of intensity at C73 in the Tsg101 UEV domain. (top-left), a view of the Tsg101 UEV domain (PDB ID: 5VKG) in gray, highlighting the location of C73, which forms a disulfide with the activated prazole in orange. Intensity plots as a function of time after prazole addition are shown for tenatoprazole and 18 tested derivatives, where loss was fit to a simple exponential decay function: $I_t = e^{-kt} + I_f$ and the values of k and I_f are given in Table 1. In addition to tenatoprazole, compounds 1, 2, 3, 6, 12, 14, 17, and 18 show significant evidence of labeling. Of these, compounds 1, 2, 12, 14, 17, and 18 have apparent rates comparable to or greater than tenatoprazole. Error bars are derived from the noise level of the spectra estimated using the Estimated Noise function in NMRPipe and NMRDraw.

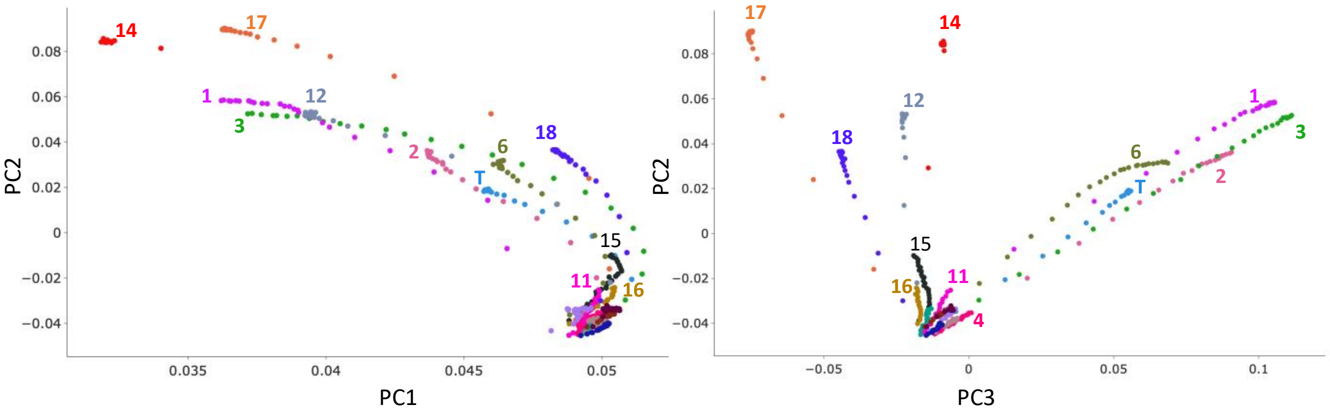

Figure S2. **Principal Component Analysis of the time-dependence of prazole-adduct formation for tested prazole derivatives shows a bifurcated relationship.** Plots of the first and second (left) and third and second (right) principal components obtained from PCA on height-normalized HSQC spectras used to follow prazole attachment. PCA results were obtained using NMRPipe and used the spectral region 7.5-9.4 ppm (^{1}H) and 112-130 ppm (^{15}N). The first two PCs separate strongly on attachment, where eg: compounds 14, 1, and 3 appear to have the greatest labeling extent over the tested interval. The third PC, however, bifurcates the compounds into two groups, where those with negative PC3 values may be further subdivided. Low values of PC3 feature the rabeprazole derivatives, where 17+18 have large PC2 values and 15+16 smaller ones. Values near zero include compounds 12+14+15, which have the intermediate linker length and biphenyls. Large PC3 values include tenatoprazole, the three smallest derivatives (1, 2, 3) and interestingly compound 6, which had the shortest linker length but with a bulky biphenyl. Separation based on PC3 appears to be tied to chemical shifts rather than intensity differences, as compounds 17 and 18 display markedly different rates and behavior at C73 while having similar PC3 coordinates.

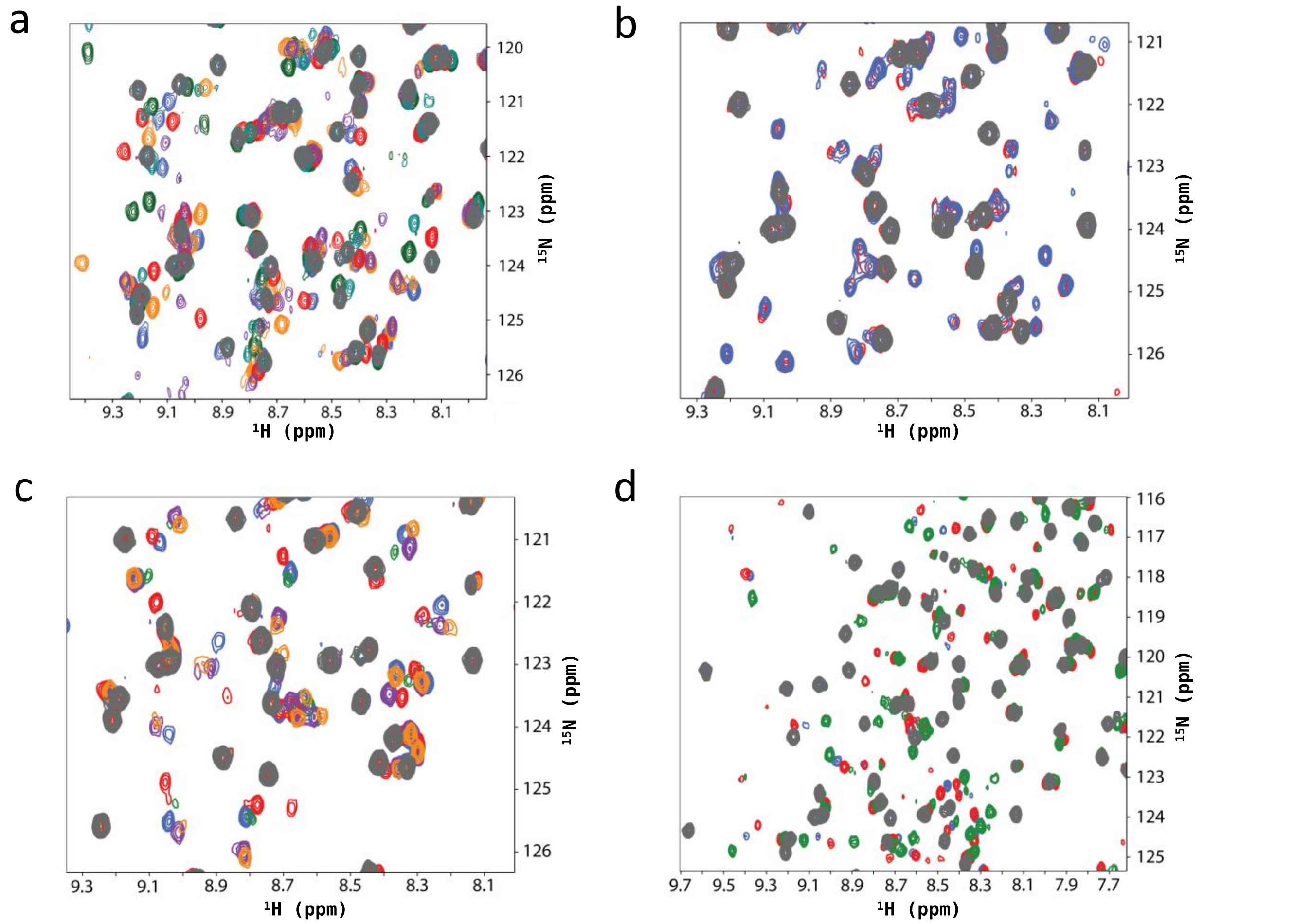

Figure S3. Overlays of clustered compounds identified in global analysis of prazole derivatives. (A) Esomeprazole (green), tenatoprazole (blue), rabeprazole (orange), ilaprazole (red), and compounds 13 (teal) and 20 (purple) form a grouping of poorly separated compounds with divergent shifts. (B) Compounds 1 (blue) and 2 (red) formed a distinct group in the third MCA dimension and displayed poor labeling and partial formation of a secondary adduct on the Tsg101 UEV domain. (C) Compounds with rabeprazole-derived linkers fall in this grouping, featuring compounds 14 (red), 15 (blue), 16 (green), 17 (purple), and 18 (orange). (D) Three compounds: 10 (blue), 11 (red), and 12 (green) with the intermediate linker length show the same apparent pose with increasing shifts (11 > 10 > 12).

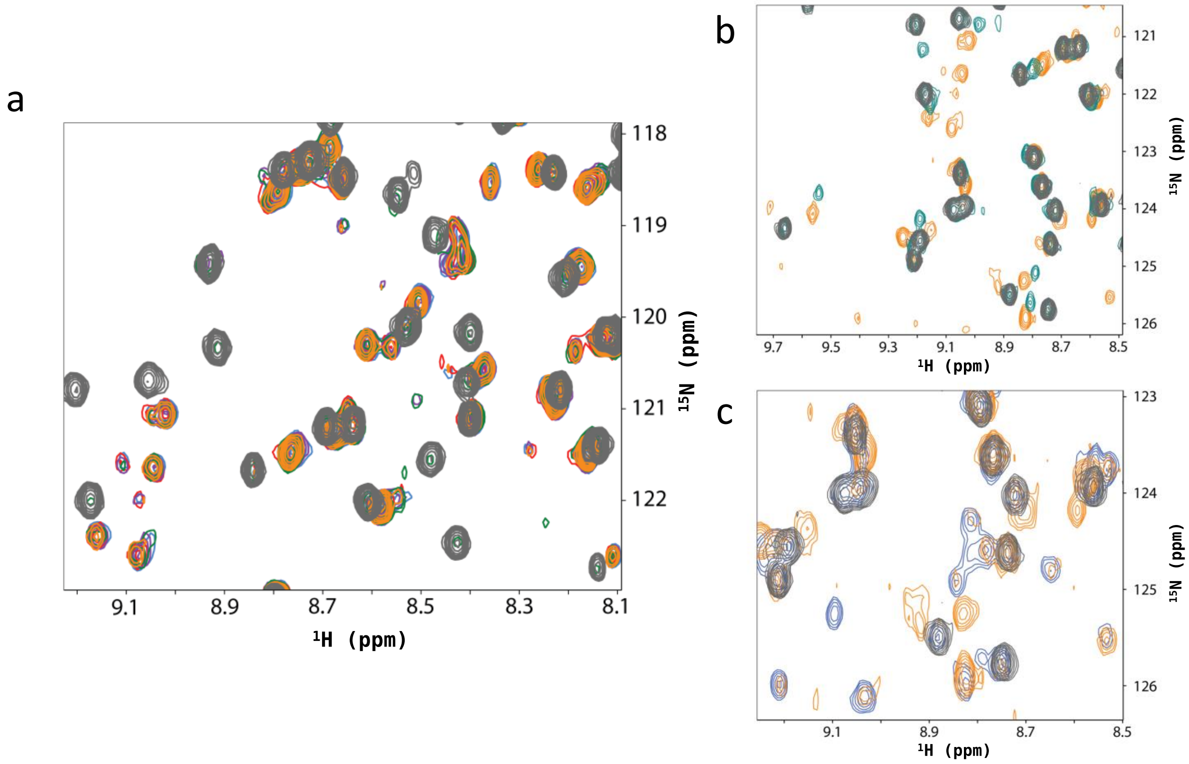

Figure S4. Overlays comparing compounds that formed secondary adducts with the Tsg101 UEV domain. (A) Five compounds: lansoprazole (lansoprazole), 3 (green), 4 (red), 6 (purple), and 7 (blue) share identical major shifts and give rise to the same secondary adduct by LC / MS, with a delta of +252 Da consistent with loss of the R3 substituent from the pyridine ring. (B) Comparison of lansoprazole (orange) with compound 13 (teal). Compound 13 also forms a secondary adduct by LC / MS, but with a mass of +238 Da, corresponding to the lack of R2 methyl relative to the other five compounds. The addition of the R2 methyl in lansoprazole appears to lead to additional interactions and multiple conformers on the UEV surface. (C) Comparison of compound 1 (blue) with an early timepoint of lansoprazole (orange), taken 7 hours after prazole addition. When labeled in PBS, compounds 1 and 2 produced evidence of multiple species which correspond poorly to the eventual major state of the secondary adduct. At this early timepoint, however, there are minor peaks that match the dominant ones seen for compound 1, indicating that the compound 1 adducts formed at high pH include an intermediate conformation or species.

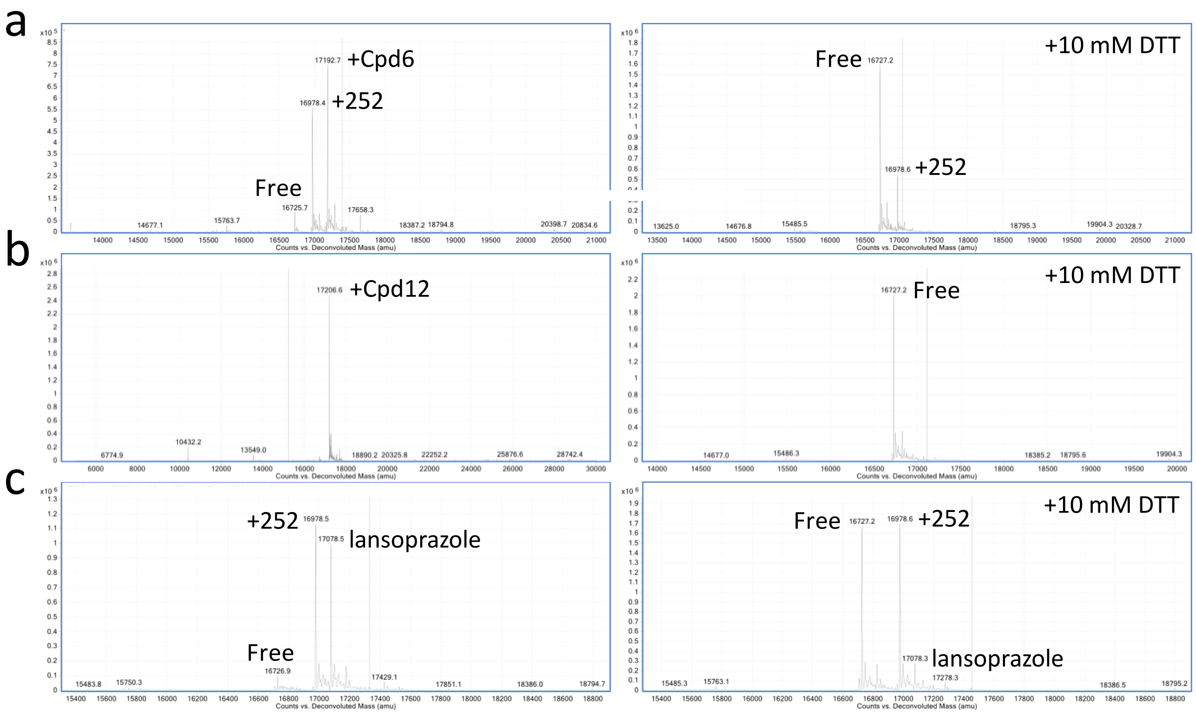

Figure S5. The secondary adduct formed for certain prazoles is irreducible. Shown are LC / MS results after labeling ^{15}N Tsg101 UEV domain with excess prazole (left) and the impact of excess DTT on the labeled adducts (right). (A) Compound 6, which has the shortest tested R3 linker length, shows approximately 90% labeling under these conditions, split between the expected (16978.4 Da) adduct mass and an anomalous +251 Da species. Addition of DTT successfully reduces the expected adduct, but the unexpected +251 Da species is retained. (B) Compound 12, which differs from 6 only in the presence of a single carbon extension in the R3 linker, shows complete labeling under these conditions to the expected (17206.6 Da) prazole adduct, which is nearly fully reduced in the presence of DTT. (C) The commercial lansoprazole compound, like compound 6 also shows the presence of both the expected adduct and the +252 Da species, where the latter once again cannot be reduced in the presence of excess DTT. Prazoles were added to 10 µM ^{15}N Tsg101 UEV in NMR buffer at 2-fold excess and incubated for 24 hours. For reduction, DTT was added to 10 mM final concentration after adjusting samples to neutral pH and incubated for 30 minutes.

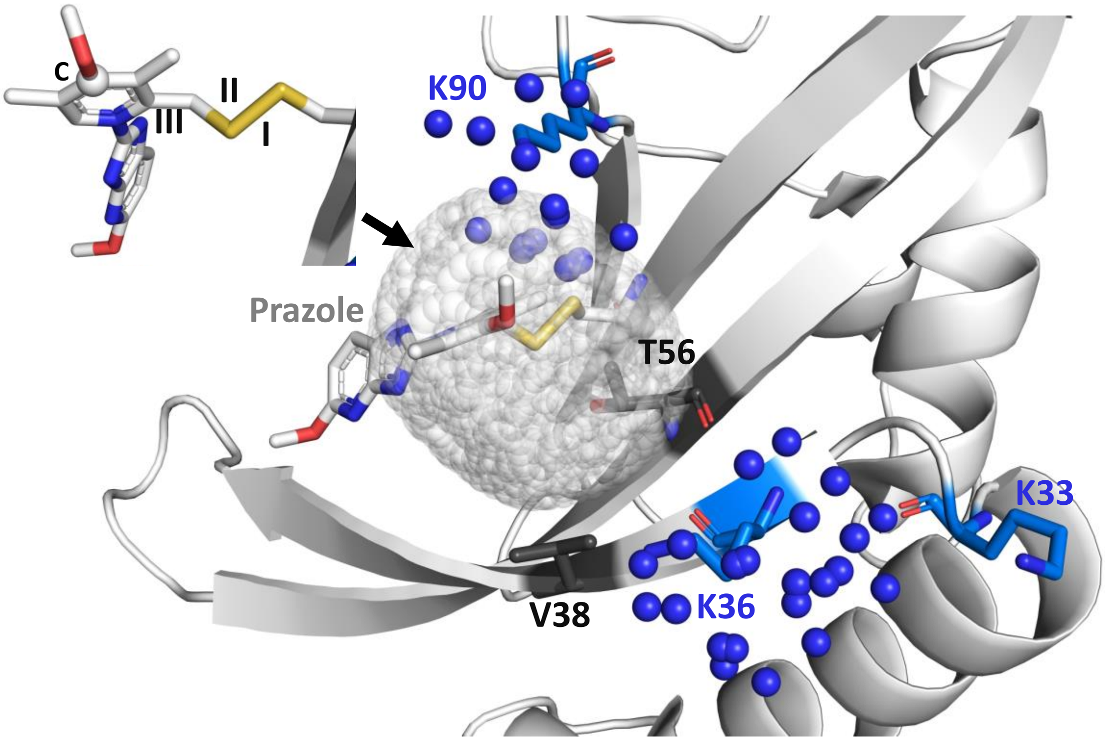

Figure S6. Modeling the Nucleophilic Environment around the Prazole bound to C73 of the Tsg101 UEV Domain. The previously determined pose (PDB ID: 5VKG) of tenatoprazole (sticks) on the surface of the Tsg101 UEV domain (gray cartoon) is shown to represent the primary adduct. The observed secondary adduct species formed with lansoprazole and several prazole derivatives involves apparent loss of the 4-pyridyl substituent, consistent with a nucleophilic attack at this position. The three closest nucleophilic residues to C73 on the Tsg101 surface are lysines 33, 36, and 90. The sidechain amine nitrogen of K33 is >1.5 nm from the 4-pyridyl carbon of the prazole, while K90 and K36 are <1 nm away. To determine which residue was likely to contribute to secondary adduct formation, we first generated the allowed K36 and K90 sidechain rotamers with the mutagenesis wizard in pymol (shown as blue spheres for the side chain nitrogen). For the prazole, rotations of the final three chi angles (I-III, inset) were sampled in 30° increments, with the cloud of resulting 4-pyridyl carbon positions shown as gray spheres. Several K90 rotamers overlap with the 4-pyridyl cloud, indicating that this position could likely be attacked by the K90 sidechain. For K36, the minimum distance between 4-pyridyl carbon and the sidechain amino nitrogen is 4.5 Å, and this orientation would likely be sterically blocked without rearrangement of other beta-sheet sidechains, including those of V38 and T56 (dark gray sticks). Thus, the most likely source of the secondary adduct is the sidechain amino nitrogen of K90.

a

Peptide	Sequence	Peptide Mass	Prazole	Expected Adduct Mass	Adduct1 Mass	Adduct2 Mass
**3mer**	GCK	347.15	Lansoprazole	698.2	698.2	598.2
**4mer**	GCGK	404.17	Lansoprazole	755.2	755.2	655.2
**5mer**	GCGGK	461.20	Lansoprazole	812.3	812.3	712.3
**6mer**	GCGGGK	518.22	Lansoprazole	869.3	869.3	769.3

b

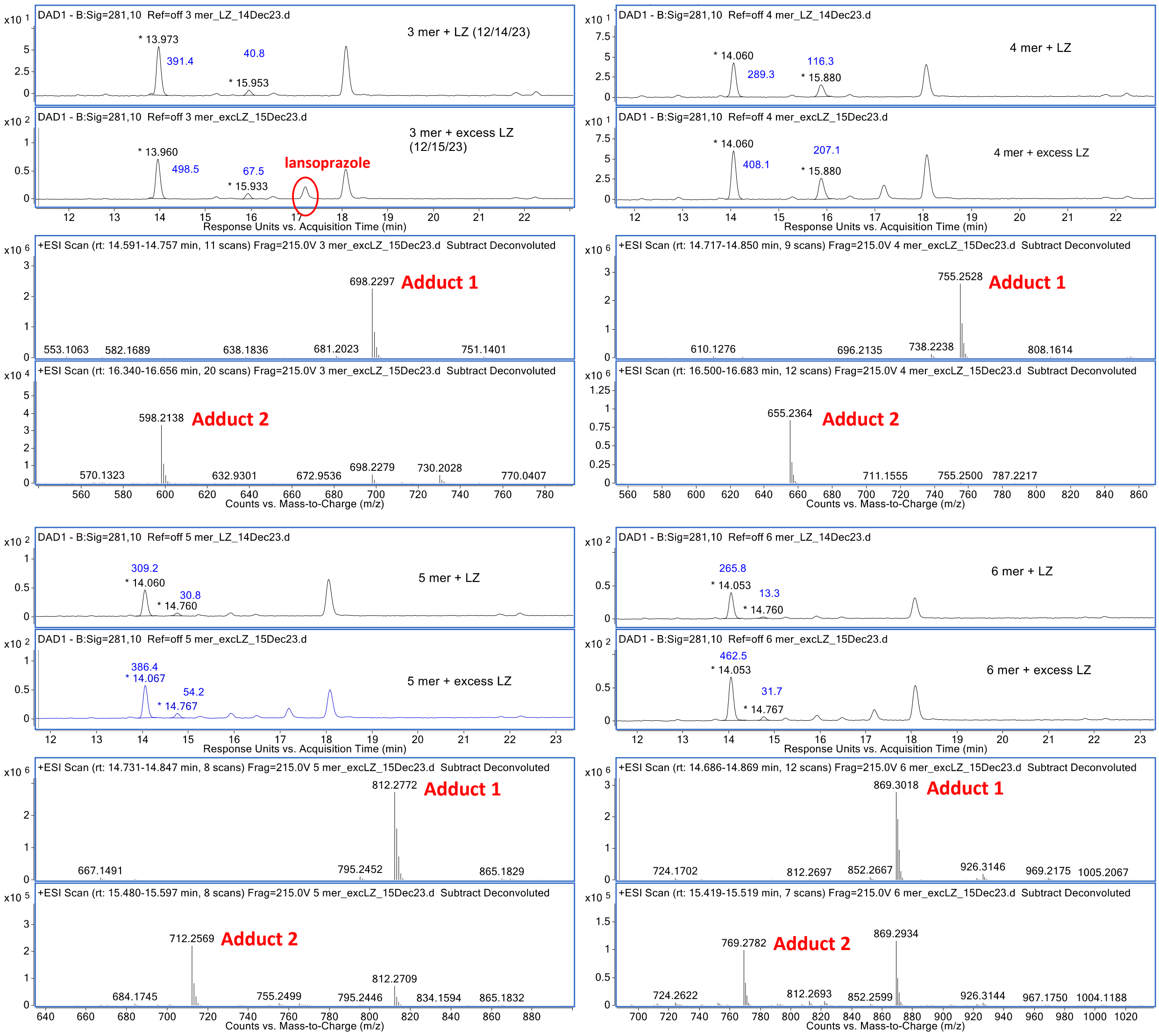

Figure S7. The Secondary Adduct observed for Tsg101 and Lansoprazole is also observed in Short CK Peptides. Having observed the formation of an irreversible, secondary adduct between Tsg101 and several prazoles, we also tested for the formation of an equivalent adduct in a simplified system having only cysteine and lysine sidechain nucleophiles. Tested peptides are given in (A), with the general sequence GCG_nK, where the expected adduct mass was taken from the observed delta mass for the primary lansoprazole adduct with Tsg101 UEV domain. All peptides were N-terminally acetylated and C-terminally amidated to prevent the previously characterized adduct formation with the peptide amino-terminus [peptide citation]. Lansoprazole was added to each peptide, where the resulting LC/MS results are given for each peptide in (B). In each case the chromatogram for the addition of stoichiometric lansoprazole is shown at top, followed by the chromatogram with 3x excess lansoprazole, and then the observed masses for peaks corresponding to the primary and secondary adducts (bottom) in the presence of excess lansoprazole. The secondary adduct is present in each case, having the same delta mass of 100 units corresponding to loss of the TFE group as seen for Tsg101 UEV domain. Compared to the protein, however, there is much less formation of the secondary adduct, with the most seen for the 4mer GCGK sequence. Reduced formation is likely reflective of the peptide flexibility reducing preferred orientations, and potential alterations in the cysteine or lysine pKas in the protein environment.

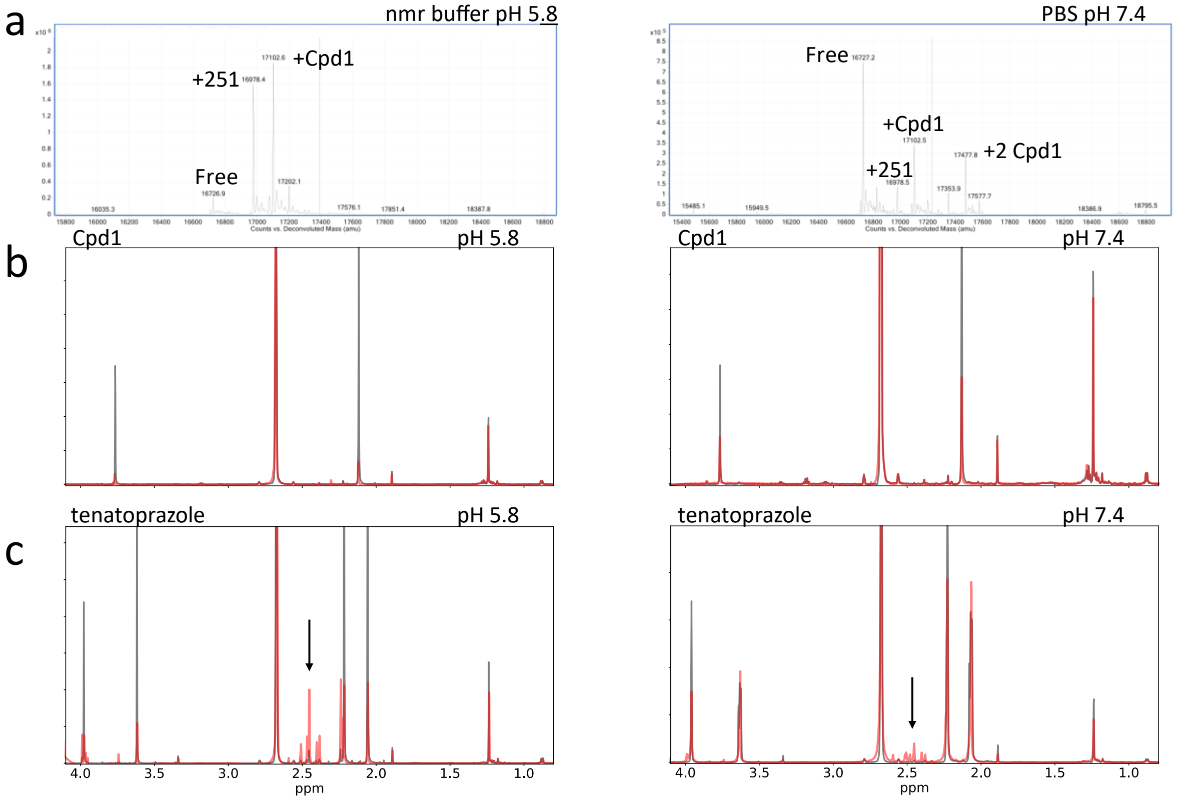

Figure S8. Small phenyl derivatives appear unstable in neutral conditions. (A) LC/MS results from labeling ^{15}N Tsg101 UEV domain with excess compound 1 in either NMR buffer (left) or PBS (right), to reproduce the conditions of the two assays. In both cases, compound 1 was added at 2-fold excess to 10 µM Tsg101 UEV domain. In the mildly acidic NMR buffer (pH 5.8), there is >90% labeling with a mixture of the expected (17102.6 Da) and secondary (16978.4) adducts. In the higher pH PBS, however, there is <50% attachment, with a mixture of species including the expected adduct at 17102.5 Da, secondary adduct at 16978.5 Da, and an apparently double-labeled species at 17477.8 Da. (B) ^{1}H NMR traces of 1 mM Compound-1 in 20% d_6-dmso and 80% NMR buffer (left) or 80% NMR buffer adjusted to pH 7.4 (right) after 30 minutes (black) and 15 hours (red). The loss of the primary, pro-drug species is slower at high pH, implying reduced conversion outside of the mildly acidic conditions, but neither condition shows secondary peaks consistent with build-up of activated drug. (C) As in (B) for tenatoprazole, highlighting the conversion to a secondary species with detectable peaks under both conditions.

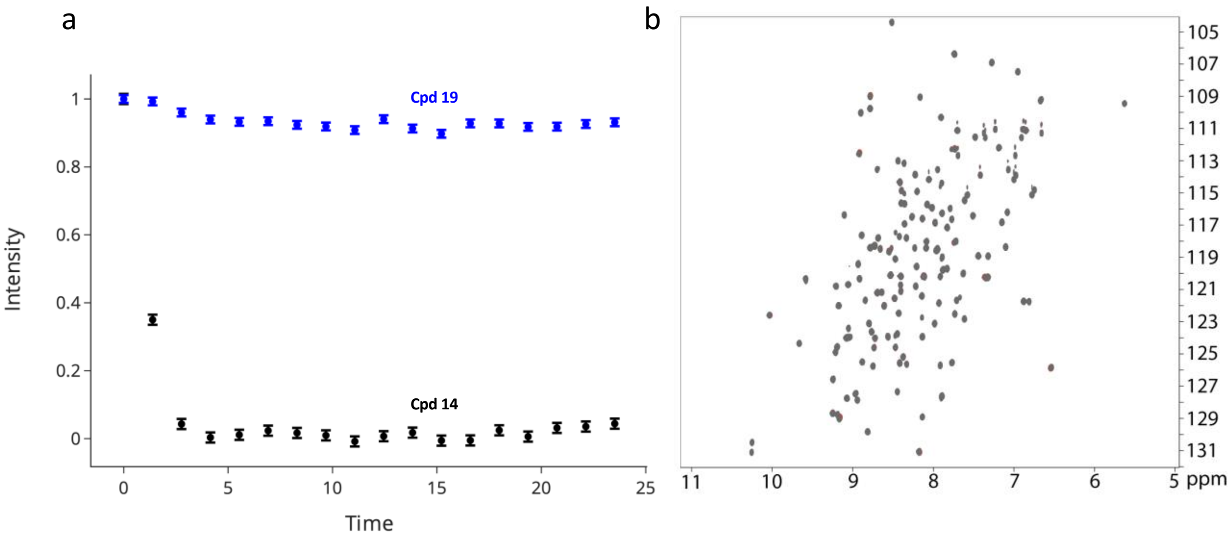

Figure S9. A sulfide prazole derivative does not form a covalent adduct with the Tsg101 UEV domain *in vitro*. (A) Comparison of the C73 intensity from 1-24 hours post-addition of stoichiometric compound 14 (black), or the sulfide variant compound 19 (blue). While Compound 14 rapidly forms a disulfide adduct with the UEV domain, evidenced by the near complete disappearance of C73 intensity by 4-6 hours, there is almost no change in intensity for compound 19 (< 5%) over the full 24-hour period. An overlay of the one (gray) and 24-hour (red) spectra also shows no evidence of covalent attachment, and a lack of minor shifts that imply minimal noncovalent affinity of the sulfide derivative for the UEV. Error bars in panel (A) are derived from the estimated noise level of the spectra obtained using the Estimate Noise function in NMRPipe and NMRDraw.

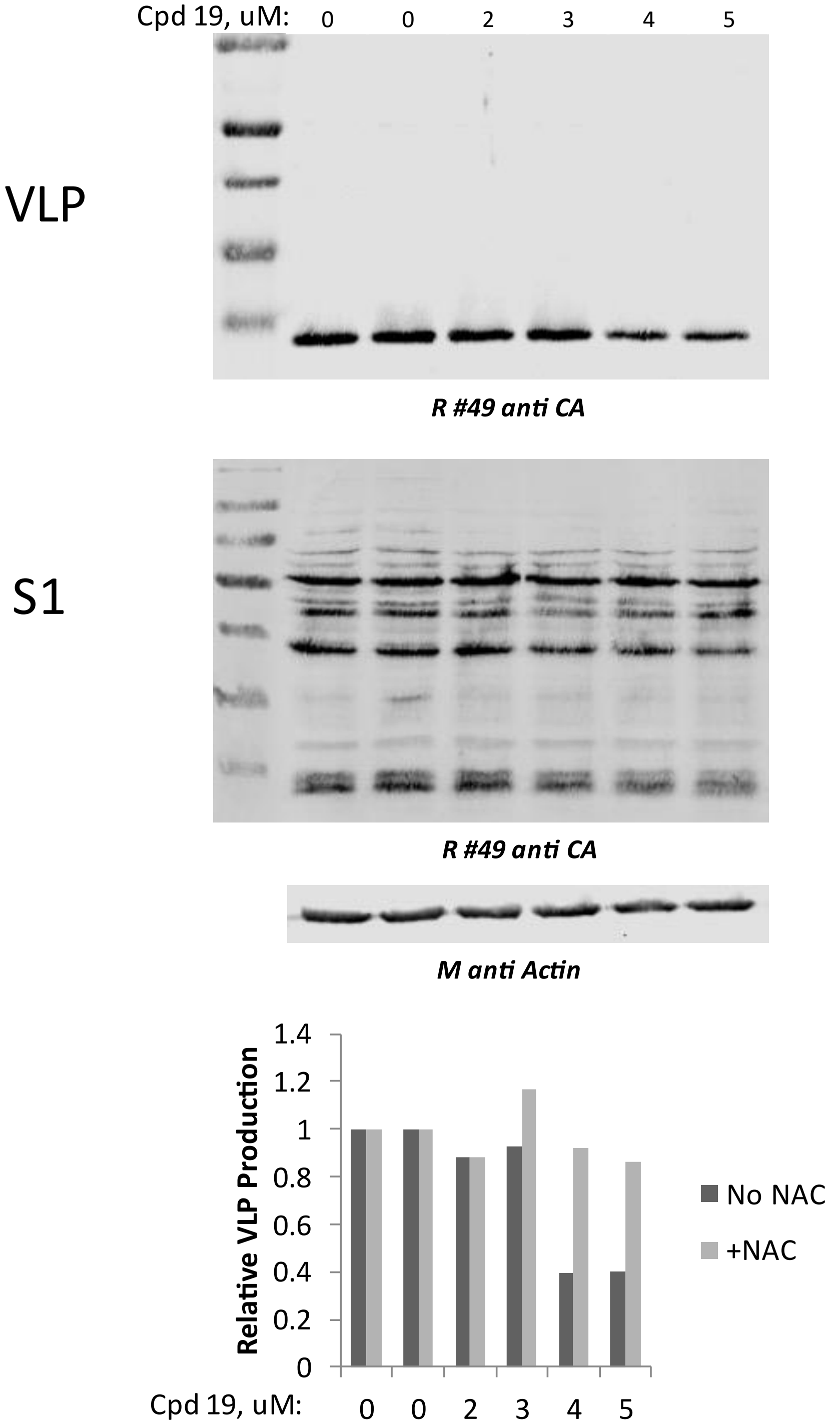

Figure S10. Prazole sulfides are sensitive to NAC competition, indicating that they act through disulfide formation within the cell. We showed previously that a N-acetyl cysteine (NAC) excess (3 mM) can compete for tenatoprazole over a broad range where the prazole is effective and suppress its ability to inhibit VLP production[5a]. Here, we show that NAC competes similarly with the sulfide derivative compound 19, indicating that its ability to inhibit must also involve Cys and, thus presumably is sulfoxidated to the sulfoxide pro-drug and converts to the same active sulfenamide.

References

[35] G. Sorba, U. Galli, C. Cena, R. Fruttero, A. Gasco, G. Morini, M. Adami, G. Coruzzi, M. I. Brenciaglia, F. Dubini, *ChemBioChem* **2003**, *4* (9), 899, https://doi.org/https://doi.org/10.1002/cbic.200300617.
[36] V. Diemer, H. Chaumeil, A. Defoin, F. Alain, A. Boeglin, C. Carre, *European Journal of Organic Chemistry - EUR J ORG CHEM* **2008**, *2008*, 1767, https://doi.org/10.1002/ejoc.200701023.

Author Contributions

D.A.N, S.W., C.C., R.S. and N.T. designed the research. R.N., V.R.S., C.M. and R.S. Synthesized the prazole derivatives. D.A.N., R.B., and N.T. performed *in vitro* characterization experiments and analyzed data. S.W. and C.C. performed HIV-1 assays and analyzed data. All authors participated in preparation and editing of the manuscript. C.C. and N.T. participated in funding acquisition for the project. N.T. supervised the project.
